# Differential Antagonism of Human Innate Immune Responses by Tick-Borne *Phlebovirus* Nonstructural Proteins

**DOI:** 10.1128/mSphere.00234-17

**Published:** 2017-06-28

**Authors:** Veronica V. Rezelj, Ping Li, Vidyanath Chaudhary, Richard M. Elliott, Dong-Yan Jin, Benjamin Brennan

**Affiliations:** a MRC—University of Glasgow Centre for Virus Research, Glasgow, Scotland, United Kingdom; b School of Biomedical Science, The University of Hong Kong, Pokfulam, Hong Kong, Special Administrative Region, China; UT Southwestern Medical Center

**Keywords:** emerging pathogens, nonstructural protein, phlebovirus, bunyavirus, innate immunity

## Abstract

In recent years, several newly discovered tick-borne viruses causing a wide spectrum of diseases in humans have been ascribed to the *Phlebovirus* genus of the *Bunyaviridae* family. The nonstructural protein (NSs) of bunyaviruses is the main virulence factor and interferon (IFN) antagonist. We studied the molecular mechanisms of IFN antagonism employed by the NSs proteins of human apathogenic Uukuniemi virus (UUKV) and those of Heartland virus (HRTV) and severe fever with thrombocytopenia syndrome virus (SFTSV), both of which cause severe disease. Using reporter assays, we found that UUKV NSs weakly inhibited the activation of the beta interferon (IFN-β) promoter and response elements. UUKV NSs weakly antagonized human IFN-β promoter activation through a novel interaction with mitochondrial antiviral-signaling protein (MAVS), confirmed by coimmunoprecipitation and confocal microscopy studies. HRTV NSs efficiently antagonized both IFN-β promoter activation and type I IFN signaling pathways through interactions with TBK1, preventing its phosphorylation. HRTV NSs exhibited diffused cytoplasmic localization. This is in comparison to the inclusion bodies formed by SFTSV NSs. HRTV NSs also efficiently interacted with STAT2 and impaired IFN-β-induced phosphorylation but did not affect STAT1 or its translocation to the nucleus. Our results suggest that a weak interaction between STAT1 and HRTV or SFTSV NSs may explain their inability to block type II IFN signaling efficiently, thus enabling the activation of proinflammatory responses that lead to severe disease. Our findings offer insights into how pathogenicity may be linked to the capacity of NSs proteins to block the innate immune system and illustrate the plethora of viral immune evasion strategies utilized by emerging phleboviruses.

**IMPORTANCE** Since 2011, there has been a large expansion in the number of emerging tick-borne viruses that have been assigned to the *Phlebovirus* genus. Heartland virus (HRTV) and SFTS virus (SFTSV) were found to cause severe disease in humans, unlike other documented tick-borne phleboviruses such as Uukuniemi virus (UUKV). Phleboviruses encode nonstructural proteins (NSs) that enable them to counteract the human innate antiviral defenses. We assessed how these proteins interacted with the innate immune system. We found that UUKV NSs engaged with innate immune factors only weakly, at one early step. However, the viruses that cause more severe disease efficiently disabled the antiviral response by targeting multiple components at several stages across the innate immune induction and signaling pathways. Our results suggest a correlation between the efficiency of the virus protein/host interaction and severity of disease.

## Abstract (precis)

Since 2011, there has been a large expansion in the number of emerging tick-borne viruses that have been assigned to the *Phlebovirus* genus. Heartland virus (HRTV) and SFTS virus (SFTSV) were found to cause severe disease in humans, unlike other documented tick-borne phleboviruses such as Uukuniemi virus (UUKV). Phleboviruses encode nonstructural proteins (NSs) that enable them to counteract the human innate antiviral defenses. We assessed how these proteins interacted with the innate immune system. We found that UUKV NSs engaged with innate immune factors only weakly, at one early step. However, the viruses that cause more-severe disease efficiently disabled the antiviral response by targeting multiple components at several stages across the innate immune induction and signaling pathways. Our results suggest a correlation between the efficiency of the virus protein/host interaction and severity of disease.

## INTRODUCTION

Phleboviruses are enveloped, single-stranded RNA viruses belonging to the *Bunyaviridae* family. The genus is comprised of over 70 viruses, broadly divided into the sandfly fever group and the Uukuniemi-like group, according to their genomic, antigenic, and vector similarities (1, 2). The viral genome is composed of the large (L), medium (M), and small (S) RNA segments. The L segment encodes the viral RNA-dependent RNA polymerase, the M segment encodes the precursor for the viral glycoproteins (Gn and Gc), and the S segment encodes the nucleocapsid (N) protein and a nonstructural protein (NSs). Viruses belonging to the sandfly fever group are transmitted by dipterans (phlebotomines and mosquitoes) and encode a nonstructural protein (NSm) at the N terminus of their glycoprotein precursor, whereas those within the Uukuniemi-like group are transmitted by ticks and do not encode an NSm protein within their genome (3, 4).

Tick-borne (TiBo) phleboviruses were not considered a public health threat until the emergence of a novel tick-borne *Phlebovirus*, named severe fever with thrombocytopenia syndrome virus (SFTSV), in China in 2009. The virus caused disease in humans, with a case fatality rate of 12% to 30%, and patients presented with fever, thrombocytopenia, hemorrhagic manifestations, and multiorgan failure (5, 6). SFTSV was later identified in patients exhibiting similar symptoms in Japan and South Korea (7, 8). Shortly after the emergence of SFTSV, a closely related virus, Heartland virus (HRTV), was isolated from patients in Missouri and Tennessee in the United States who presented with fever, fatigue, thrombocytopenia, leukopenia, and, in some cases, multiorgan failure and hemorrhage (9–11). Unlike highly pathogenic TiBo phleboviruses, some viruses, such as the prototype Uukuniemi virus (UUKV), are apathogenic. While antibodies against UUKV-like viruses have been detected in birds, rodents, cows, and humans (12, 13), no disease of medical or veterinary significance has been associated with UUKV infection (14). In recent years, many novel and emerging viruses have been assigned to the *Phlebovirus* UUKV-like group, including Lone Star virus (LSV) (15), Hunter Island group virus (HIGV) (16), Malsoor virus (MALV) (17), Antigone virus (ANTV) (18), blacklegged tick *Phlebovirus* (BTPV), and American dog tick *Phlebovirus* (ADTPV) (19). The continuing expansion of the host and geographical ranges of tick-borne phleboviruses poses a potential risk to both human and animal health.

Following infection of a susceptible host, viruses confront the innate immune system, the first line of defense against viral infections. RNA viruses produce products such as double-stranded RNA (dsRNA) and 5′-triphosphorylated uncapped single-stranded RNAs (ssRNAs) during replication of their viral genome. These products, or pathogen-associated molecular patterns (PAMPs), are detected by host cell RNA helicases such as those encoded by melanoma differentiation-associated gene 5 (MDA-5) and retinoic acid-inducible gene I (RIG-I), respectively (20). As some negative-strand RNA viruses produce little or undetectable amounts of dsRNA during replication (21, 22), it is hypothesized that these viruses are sensed mainly by RIG-I, through the generation of single-stranded RNA (ssRNA) with uncapped 5′ triphosphate ends (23, 24). Binding of viral RNA to RIG-I results in its activation and the initiation of downstream signaling pathways. Activated RIG-I can recruit the adaptor mitochondrial antiviral signaling protein (MAVS, also known as IPS-1, Cardif, or VISA) through caspase activation and recruitment domains (CARD), which leads to the subsequent activation of interferon (IFN) regulatory factor-3 (IRF-3), IRF-7, and NF-κB via kinases TBK1/IκB kinase-ε (TBK1/IKKε) and IKKα/IKKβ, respectively. Activated IRF-3 and NF-κB can then translocate to the nucleus and act as transcription factors for the initiation of beta interferon (IFN-β) mRNA synthesis (25, 26). Following IFN induction, secreted IFN activates the IFN signaling pathway in neighboring cells by binding to IFN receptors, triggering the activation of the JAK/STAT pathway. Type I IFN signaling results in the formation of the heterotrimer ISGF3, composed of STAT1, STAT2, and IRF-9, which binds to the interferon-stimulated response element (ISRE) and enhances transcription of numerous antiviral interferon-stimulated genes (ISGs) (27).

Bunyaviruses have evolved countermeasures to evade the host innate immune system. The NSs protein is a nonessential protein encoded by some members of the *Bunyaviridae* and has been shown to contribute to virus virulence and pathogenesis by acting as an IFN antagonist (28, 29). Within the *Phlebovirus* genus, studies on the IFN antagonist activity of NSs proteins have focused on mosquito-borne phleboviruses, in particular, that encoded by Rift Valley fever virus (RVFV). With the emergence of highly pathogenic SFTSV, research interests have focused on understanding its underlying molecular mechanisms of pathogenicity. As a result, quickly after its emergence, SFTSV NSs was characterized as a potent antagonist of IFN induction and signaling, through the spatial isolation of TRIM25 and RIG-I (30), TBK1 (31, 32), IRF-3 (31, 32), and STAT1 and STAT2 (33, 34) in NSs-containing, round, cytoplasmic inclusion bodies (IB) or viroplasms. Although the interaction between SFTSV NSs and IKKε or IRF-3 was reported to be indirect, facilitated through the interaction between SFTSV NSs and TBK1 (32), one study also showed a direct interaction of SFTSV NSs with IKKε (31). Similarly, while one study reported a direct interaction between SFTSV NSs and RIG-I (30), no direct interaction was observed in another study (31). We have previously shown that UUKV NSs also acts as an IFN antagonist (35). However, little is known about the mechanism by which UUKV NSs and other tick-borne *Phlebovirus* NSs proteins impair the innate immune system.

Here we report a study of UUKV and HRTV NSs proteins, focused on understanding their role in antagonizing the human innate immune system at the molecular level, in comparison to the well-characterized SFTSV NSs. We observed that UUKV NSs weakly suppresses IFN induction but not IFN signaling, compared to the potent effects of HRTV and SFTSV NSs in both pathways. Our findings indicate that the weak suppression of IFN induction by UUKV NSs occurs through a direct interaction with MAVS, an early effector protein in the RIG-I signaling pathway. The weak effects of UUKV NSs as an IFN antagonist may provide an explanation for its inability to cause disease in humans, which is in agreement with previous studies (35). For the first time, we show that HRTV NSs acts as a potent antagonist of IFN induction and type I IFN signaling. Moreover, we observed that despite HRTV and SFTSV NSs proteins sharing only 63% amino acid identity (5), some key characteristics of their IFN inhibitory activity are conserved. Our data show that both proteins interact with TBK1 and antagonize its phosphorylation at serine 172 to interfere with IFN induction and with STAT2 to block its phosphorylation and thus interfere with type I IFN signaling. HRTV NSs showed diffused cytoplasmic localization, unlike the inclusion bodies formed by SFTSV NSs to spatially isolate its interacting partners in the IFN induction and signaling pathways. Additionally, we demonstrate that type II IFN signaling is not inhibited by HRTV or SFTSV NSs proteins, presumably due to a weak or indirect interaction of the NSs proteins with STAT1. The inability of HRTV and SFTSV NSs proteins to efficiently block type II IFN signaling might explain the activation of proinflammatory responses that lead to severe disease following virus infection.

The findings presented here expand our knowledge of phleboviral strategies to modulate host innate immune responses and illustrate that TiBo *Phlebovirus* NSs proteins can evolve divergent mechanisms to antagonize the IFN system.

## RESULTS

### Diversity of TiBo *Phlebovirus* NSs subcellular localization.

The NSs protein of bunyaviruses is known to exhibit very low conservation at the amino acid level in comparison to other viral proteins (5, 36). Therefore, we speculated that the differences in amino acid sequence could permit the NSs proteins to adopt different IFN antagonism strategies. To begin a basic characterization of tick-borne *Phlebovirus* NSs proteins, we first compared the amino acid identities of UUKV (GenBank accession no. AAA47959.1), HRTV (GenBank accession no. AFP33392.1), and SFTSV (GenBank accession no. AJD86041.1) NSs proteins using CLUSTAL Omega (37, 38) (Table 1). While UUKV NSs shares low amino acid sequence identity with HRTV (22.73%) and SFTSV (22.22%) NSs, the identity between HRTV and SFTSV NSs proteins is 62.76% (Table 1). Alignments of the amino acid sequences of NSs proteins relevant to this study are shown in Fig. S1 in the supplemental material.

10.1128/mSphere.00234-17.1 FIG S1  Amino acid sequence alignment of UUKV, HRTV, and SFTSV NSs. UUKV, HRTV, and SFTSV NSs amino acid sequences (GenBank sequences AAA47959.1, AFP33392.1, and AJD86041.1, respectively) were aligned using CLUSTAL Omega, and the alignment was visualized with Jalview 2 (100). Residues are highlighted in blue according to the percentage of identity relative to the consensus sequence (dark blue, higher percentage of identity; light blue, lower percentage of identity). The conservation histogram below the alignment depicts conservation scores for each position (0 to 10; an asterisk [*] refers to a score of 11, indicating conserved amino acids). The PxxP motif found in HRTV and SFTSV NSs amino acid sequences is highlighted (red box). Download FIG S1, TIF file, 10 MB. Copyright © 2017 Rezelj et al. 2017 Rezelj et al. This content is distributed under the terms of the Creative Commons Attribution 4.0 International license.

**TABLE 1:** Percentages of amino acid identity of tick-borne *Phlebovirus* proteins used in this study

Virus	Amino acid identity (%)^a^		
	UUKV	HRTV	SFTSV
UUKV	100	22.73	22.22
HRTV	22.73	100	62.67
SFTSV	22.22	62.67	100

a Values are based on data from GenBank (accession numbers AAA47959.1, AFP33392.1, and AJD86041.1 for UUKV, HRTV, and SFTSV NSs, respectively) and were generated using Clustal Omega.

**FIG 1:**
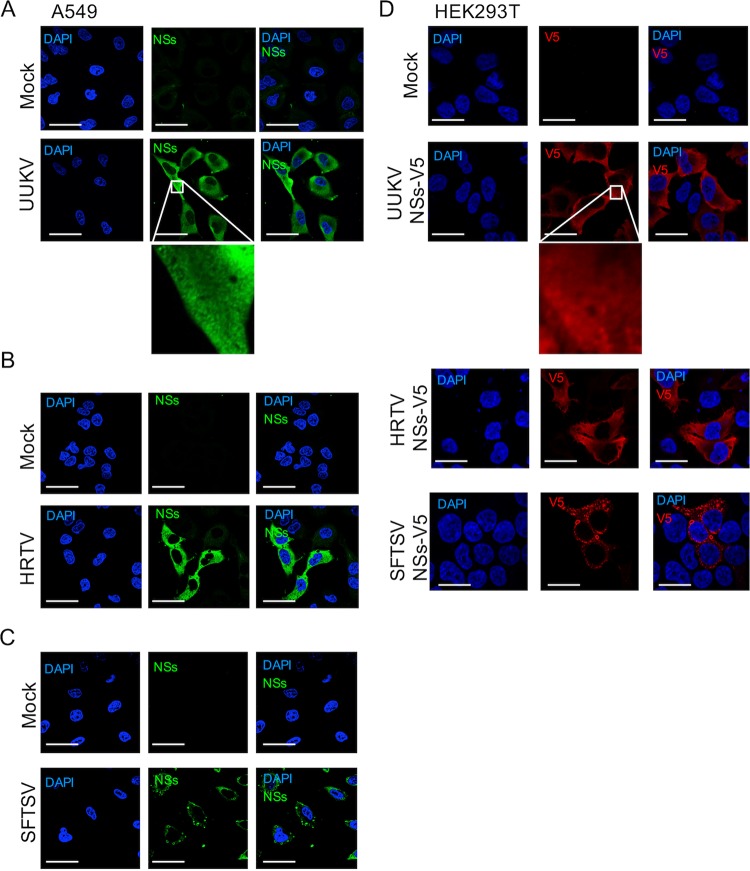
Subcellular localization of UUKV, HRTV, and SFTSV NSs. The subcellular localization of UUKV (A), HRTV (B), or SFTSV (C) NSs proteins was analyzed by confocal microscopy. The indicated cell lines were infected with UUKV, HRTV, and SFTSV at an MOI of 3 FFU/cell (or were mock infected), fixed at 24 h p.i., permeabilized, and probed with the appropriate NSs antibody. NSs proteins (green) and cell nuclei (blue) were visualized by confocal microscopy. (D) The subcellular localization of V5-tagged NSs proteins was also investigated. HEK293T cells were transfected with plasmids encoding UUKV, HRTV, or SFTSV NSs proteins tagged at their C terminus with a V5 tag. At 24 h posttransfection, cells were fixed, permeabilized, and probed with an anti-V5 antibody. DAPI-stained nuclei (blue) and the V5-tagged NSs proteins (red) were visualized by confocal microscopy. Scale bars indicate 20 μm.

We next compared the subcellular localization of the NSs proteins of UUKV and HRTV to that of SFTSV to give us insights into whether the mechanisms of IFN antagonism between these proteins are divergent or conserved. Human A549 cells were infected at a high multiplicity of infection (MOI) (3 focus-forming units [FFU]/cell) and probed with the respective NSs antibodies 24 h postinfection (p.i.). In agreement with previous reports (39), we observed cytoplasmic distribution of UUKV NSs with small, punctate staining (Fig. 1A). Interestingly, despite the higher percentage of identity between HRTV and SFTSV NSs, we noted that HRTV NSs showed diffused cytoplasmic distribution, whereas SFTSV NSs formed characteristic inclusion bodies (Fig. 1B and C). On the basis of these observations, our findings suggested that UUKV NSs and HRTV NSs may utilize a mechanism different from that of the well-studied SFTSV NSs to circumvent the IFN response.

UUKV, HRTV, and SFTSV NSs proteins, which were tagged at their C terminus with a V5 tag, were used in the rest of this study to enable a comparison of the expression levels of the tagged-NSs proteins in transient-expression assays. Thus, we additionally compared the subcellular localization of the V5-tagged NSs proteins to that of native NSs proteins following virus infection. Expression of V5-tagged UUKV, HRTV, or SFTSV NSs in HEK293T cells revealed that their subcellular localization was like that described for wild-type (wt) protein following virus infection in A549 cells (Fig. 1D).

### Inhibition of IFN induction by TiBo *Phlebovirus* NSs proteins.

**FIG 2:**
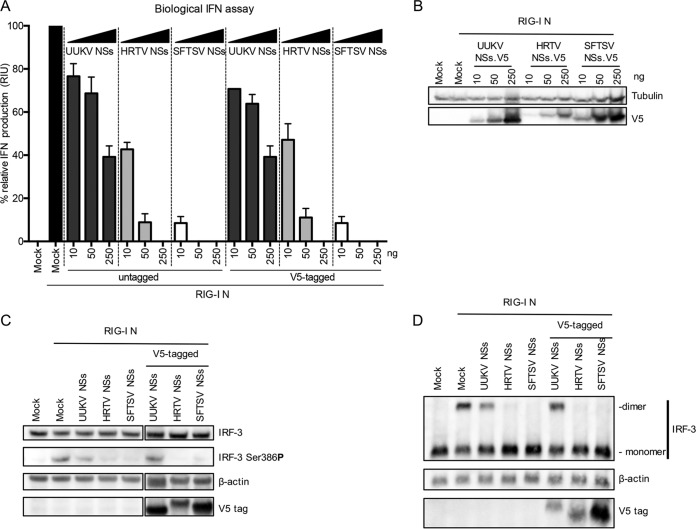
UUKV, HRTV, and SFTSV NSs proteins inhibit IFN induction. (A) The inhibitory effect of UUKV, HRTV, and SFTSV NSs proteins on IFN induction was investigated by measuring the RIG-I N-induced induction of IFN production in HEK293T cells in the presence of increasing amounts of untagged or V5-tagged NSs proteins. Cell culture supernatant was harvested 24 h posttransfection, and relative IFN units were quantified by a biological IFN assay in A549/BVDV-Npro cells. Induction was normalized against the induction control, whose results were assigned a value of 100%. The data represent results of three independent experiments performed in duplicate (*n* = 6), presented as fold induction means ± SEM. (B) Western blot of V5-tagged NSs proteins in the biological IFN assay. (C) Western blot showing phosphorylation of IRF3 at Ser386 in HEK293T cells transfected with 250 ng untagged or V5-tagged UUKV, HRTV, or SFTSV NSs proteins and induced by cotransfection of a plasmid encoding the N terminus of RIG-I. Blots shown for IRF-3, β-actin, and the V5 tag were blotted on the same membrane, with two lanes removed (indicated by the separation in the figure; uncropped images can be found in Fig. S4). (D) Western blot of monomeric and dimeric forms of IRF-3 in HEK293T cells cotransfected with 250 ng untagged or V5-tagged UUKV, HRTV, or SFTSV NSs proteins and induced by cotransfection of a plasmid encoding the N terminus of RIG-I. At 24 h posttransfection, the cell monolayer was lysed with nonreducing lysis buffer and the proteins were separated on a nonreducing gel before Western blotting and probing with IRF-3, actin, and the V5 tag antibodies was performed.

We investigated whether these NSs proteins could inhibit the IFN response at the level of IFN induction. Briefly, HEK293T cells were cotransfected with an inducer plasmid encoding the N-terminal 2CARD domain of RIG-I (pCAGGS-FLAG-2CARD [a kind gift from B. Hale, University of Zurich], here referred to as RIG-I N) and either untagged or V5-tagged UUKV, HRTV, or SFTSV NSs. V5-tagged proteins were used to compare expression levels of proteins in the assays. At 24 h posttransfection (p.t.), IFN production in these cells was quantified using a biological IFN assay. A dose-dependent inhibitory effect of IFN induction was observed for both wt and V5-tagged NSs proteins of all three viruses (Fig. 2A). However, the inhibitory activity of UUKV NSs with respect to IFN induction was distinctly weak compared to that seen with HRTV or SFTSV NSs. At the highest dose (250 ng) of NSs-encoding plasmid, UUKV NSs expression resulted in approximately 60% inhibition of IFN production, whereas that of HRTV and SFTSV NSs was 100% (Fig. 2A). This observation agrees with our recent finding that UUKV NSs acts as a weak IFN antagonist, shown by the creation of a recombinant UUKV lacking NSs (35). HRTV and SFTSV NSs proteins strongly antagonized the production of IFN (Fig. 2A). A difference in the levels of efficiency with which HRTV and SFTSV NSs can block IFN production was observed. While an approximately 50% decrease in relative IFN production was observed at the lowest dose of HRTV NSs (10 ng), this dose of SFTSV NSs resulted in an almost 95% decrease in IFN production (Fig. 2A). However, examination of the cell lysates by Western blotting revealed V5-tagged HRTV NSs expression levels that were visibly lower than those seen with SFTSV and UUKV NSs proteins (Fig. 2B). In fact, transfection of 50 ng of V5-tagged HRTV NSs resulted in expression levels similar to those seen with 10 ng of V5-tagged SFTSV NSs (Fig. 2B), and the same level of inhibition of IFN production was observed with these respective amounts (Fig. 2A). Regardless, these results indicated that all NSs proteins examined were capable of inhibiting the induction of IFN.

The transcription factor interferon regulatory factor 3 (IRF-3) is ubiquitously expressed in cells. Following activation of the IFN pathway, IRF-3 is phosphorylated by TANK-binding kinase-1 (TBK1), which allows its homodimerization prior to translocation to the nucleus (40–43). Thus, we investigated whether the NSs proteins inhibited the induction of IFN by ultimately preventing the activation of IRF-3. HEK293T cells were induced with a RIG-I N-encoding plasmid in the presence of 250 ng of untagged or V5-tagged NSs proteins and were subsequently harvested for analysis of IRF-3 phosphorylation. IRF-3 phosphorylated at Ser386 could be readily detected in the mock cells stimulated with RIG-I N and also in the presence of UUKV NSs (Fig. 2C). Almost undetectable levels of phosphorylated IRF-3 were observed in the presence of HRTV NSs or SFTSV NSs. Under all experimental conditions, the levels of total IRF-3 remained unchanged. Similarly, when cell lysates were analyzed under nondenaturing conditions, the dimeric form of IRF-3 could be detected only in the presence of UUKV NSs but not HRTV or SFTSV NSs. A shift in molecular weight was observed for the V5-tagged UUKV NSs under nonreducing conditions (Fig. 2D).

Taken together, these data indicate that, in contrast to UUKV NSs, HRTV and SFTSV NSs proteins can efficiently antagonize IFN induction, ultimately hindering the activation of IRF-3 and thus blocking activation of the IFN-β promoter. On the other hand, UUKV NSs, a weak IFN antagonist, is unable to completely inhibit the activation of IRF-3 and the subsequent production of IFN.

### UUKV NSs exerts its IFN-antagonistic activity at the level of MAVS, while HRTV NSs antagonizes at the level of TBK1.

**FIG 3:**
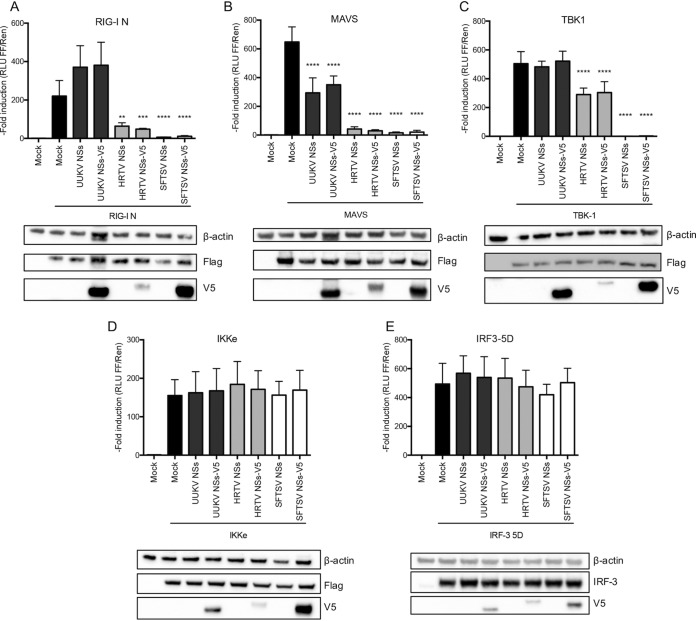
UUKV NSs protein inhibits IFN-β induction at the level of MAVS, whereas HRTV NSs antagonizes at the level of TBK1. (A to E) HEK293T cells were cotransfected with a Firefly luciferase reporter plasmid under the control of an IFN-β promoter and a *Renilla* luciferase control plasmid in the presence of untagged or V5-tagged UUKV, HRTV, or SFTSV NSs proteins. The cells were stimulated by cotransfection of inducer plasmids encoding FLAG-tagged RIG-I N (A), MAVS (B), TBK1 (C), or IκB kinase ε (IKKε) (D) or by cotransfection of a plasmid expressing untagged IRF3-5D (E). Fold induction was obtained by normalizing the luciferase values to the nonstimulated mock control sample values. Western blots of transfected cell lysates are shown in the lower panels. The data represent results of three independent experiments performed in duplicate (*n* = 3), presented as fold induction means ± SD. Statistical significance for the comparison of means between groups was determined by one-way ANOVA followed by Dunnett's multiple-comparison *post hoc* tests. ****, *P* ≤ 0.0001; ***, *P* ≤ 0.001; **, *P* ≤ 0.01; *, *P* ≤ 0.05.

To elucidate the stage of the IFN induction pathway at which UUKV and HRTV NSs proteins antagonize this response, we performed an IFN-β reporter assay in the presence of UUKV or HRTV NSs, using SFTSV NSs as a control. Various effectors of the IFN induction pathway (constitutively active RIG-I [RIG-I N], MAVS, TBK1, IKKε, or a phosphomimetic, active form of IRF-3 [IRF-3 5D]) were overexpressed in HEK293T cells to examine the activation of the IFN-β promoter in the presence of untagged or V5-tagged UUKV, HRTV, or SFTSV NSs. For UUKV NSs, inhibition was observed only in the MAVS induction reporter assay (Fig. 3B) and not in the RIG-I N, TBK1, IKKε, or IRF-3 5D induction assays (Fig. 3A and C to E). As RIG-I is involved in the activation of MAVS, the lack of inhibition by UUKV NSs at the level of RIG-I (Fig. 3A) despite its inhibition of MAVS-induced IFN-β promoter activation (Fig. 3B) was surprising. It is possible that the overexpression of RIG-I N allows the detection of an inhibitory effect of UUKV NSs on IFN production in a biological IFN assay (Fig. 2A) whereas measurement of luciferase activity upon the transactivation of the murine IFN-β promoter saturates the weak inhibitory effect of UUKV NSs at the level of RIG-I N (Fig. 3A). A small increase in IFN production was detected upon transfection of RIG-I N and UUKV NSs or NSs-V5; however, this increase was not significant and was within the limits of experimental variation within the system.

Transactivation of the IFN-β promoter was inhibited by HRTV NSs and SFTSV NSs upon stimulation by RIG-I N, MAVS, and TBK1 (Fig. 3A to C) but not by downstream factors IKKε and IRF-3 5D (Fig. 3D and E). While 99.7% inhibition of TBK1-induced activation of the IFN-β promoter by untagged and V5-tagged SFTSV NSs was observed, only 43% inhibition by untagged and V5-tagged HRTV NSs was observed (Fig. 3C). Of note, the difference in the levels of efficiency of antagonism of TBK1-induced IFN-β promoter activation may be explained by the fact that the levels of HRTV NSs expression were lower than the levels of SFTSV NSs expression, as detected by Western blotting of transfected cell lysates (Fig. 3C). Our data suggest that UUKV NSs inhibits IFN production at the level of MAVS, whereas HRTV NSs inhibits RIG-I N-, MAVS-, and TBK1-induced IFN-β reporter activity.

### UUKV NSs antagonizes IFN induction through a direct interaction with MAVS.

**FIG 4:**
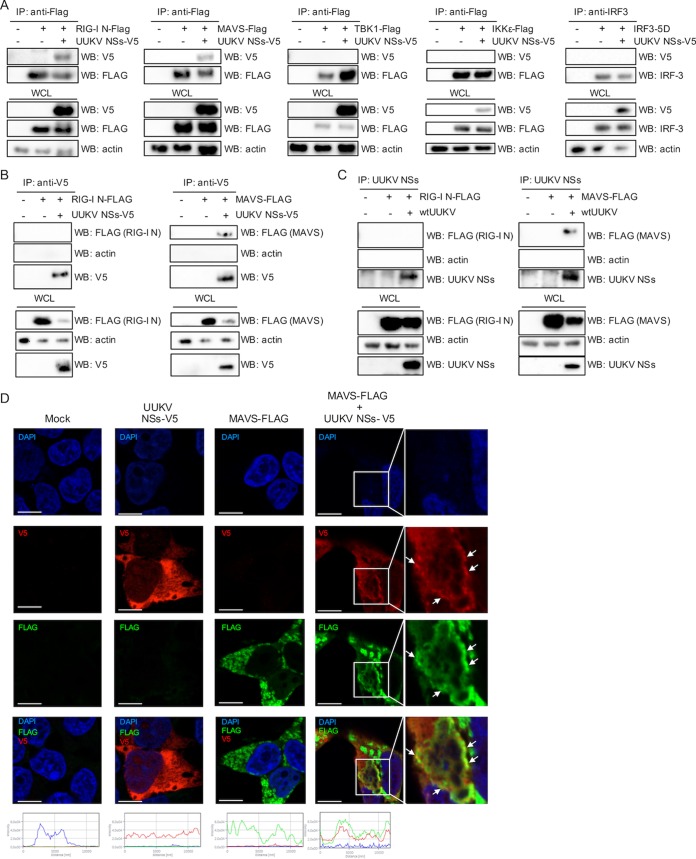
UUKV NSs interacts with MAVS. (A) HEK293T cells were cotransfected with a V5-tagged UUKV NSs-encoding plasmid along with FLAG-tagged RIG-I (N) MAVS, TBK1, IKKε, or untagged IRF3-5D-encoding plasmids. Transfected cell lysates were subjected to coimmunoprecipitation (co-IP) with beads conjugated to FLAG or IRF3 antibodies. V5-tagged UUKV NSs was detected through Western blotting with an anti-V5 antibody. (B) Reverse co-IP. HEK293T cells were mock transfected or transfected with V5-tagged UUKV NSs and FLAG-tagged RIG-I N (left panel) or MAVS (right panel). Cell lysates were subjected to co-IP with an anti-V5 antibody. FLAG-tagged RIG-I N and MAVS in the co-IP eluates were detected by Western blotting with an anti-FLAG antibody. (C) co-IP of UUKV NSs upon UUKV infection in the presence of FLAG-tagged RIG-I N or MAVS. HEK293T cells were mock infected or infected with UUKV at a high MOI (20 FFU/cell) for 8 h, followed by mock transfection or transfection of FLAG-tagged RIG-I N-encoding plasmids (left panel) or MAVS-encoding plasmids (right panel). At 30 h p.i., the cell lysates were subjected to co-IP using an anti-UUKV NSs antibody, followed by detection of FLAG-tagged RIG-I N or MAVS and UUKV NSs by Western blotting. (D) Subcellular localization of UUKV NSs-V5 and FLAG-tagged MAVS. HEK293T cells were mock transfected or transfected with UUKV NSs-V5 or FLAG-tagged MAVS or both. At 24 h posttransfection, the cells were fixed, permeabilized, and probed with V5 (red) and FLAG (green) antibodies. DAPI-stained nuclei (blue) and subcellular localization of the proteins were analyzed by confocal microscopy. Intensity profile graphs are shown at the bottom of the image (*x* axis, distance [in micrometers]; *y* axis, intensity). Scale bars indicate 10 μm. IP, immunoprecipitation; WB, Western blotting; WCL, whole-cell lysate.

We investigated whether the ability of UUKV NSs to inhibit IFN induction was due to a direct interaction with effectors of the IFN induction pathway. HEK293T cells were cotransfected with V5-tagged UUKV NSs and each of the FLAG-tagged IFN induction components or untagged IRF-3 5D, and the cell lysates were subjected to coimmunoprecipitation (co-IP) using anti-FLAG or anti-IRF-3 antibodies 18 h posttransfection. UUKV NSs was coimmunoprecipitated only with FLAG-tagged RIG-I N and MAVS and not with TBK1, IKKε, or IRF-3 5D (Fig. 4A). As a control, we confirmed that no V5-tagged NSs was pulled down from transfected cells during co-IP using anti-FLAG beads or anti-IRF-3 antibody (Fig. S2). RIG-I can interact with MAVS through its N-terminal 2CARD domain (44, 45). Thus, to ensure that the pulldown of UUKV NSs with RIG-I N or MAVS was not due to indirect interactions, we carried out reverse co-IPs. HEK293T cells transiently expressing V5-tagged UUKV NSs and FLAG-tagged RIG-I N or MAVS were subjected to co-IP with an anti-V5 antibody. Under these conditions, FLAG-tagged MAVS, but not FLAG-tagged RIG-I N, could be coimmunoprecipitated in the presence of V5-tagged UUKV NSs (Fig. 4B). These results were also confirmed in the context of UUKV infection. HEK293T cells were infected with UUKV at a high MOI (20 FFU/cell) for 8 h, followed by transient expression of FLAG-tagged RIG-I N or MAVS. At 30 h p.i., cell lysates were subjected to immunoprecipitation using an anti-UUKV NSs antibody. While no RIG-I N was coimmunoprecipitated with UUKV NSs (Fig. 4C, left panel), a weak band was observed for MAVS (Fig. 4C, right panel). The detection of a weak signal for MAVS (detectable only upon long exposure of the membrane), relative to the expression levels detected in the whole-cell lysate, suggested a weak interaction between UUKV NSs and MAVS.

10.1128/mSphere.00234-17.2 FIG S2  Control immunoprecipitation reactions to confirm lack of nonspecific binding of V5-tagged NSs proteins in co-IP experiments. Cell lysates from HEK293T cells transfected with V5-tagged UUKV, HRTV, or SFTSV NSs were immunoprecipitated with anti-FLAG beads (A) or IRF3 antibody (B) to confirm that no V5-tagged NSs protein was pulled down. Download FIG S2, TIF file, 3.7 MB. Copyright © 2017 Rezelj et al. 2017 Rezelj et al. This content is distributed under the terms of the Creative Commons Attribution 4.0 International license.

Next, we examined whether UUKV NSs and MAVS could colocalize to confirm our co-IP and reporter assay data. We overexpressed FLAG-tagged MAVS together with V5-tagged UUKV NSs in HEK293T cells. Following its activation, MAVS has been reported to adopt a speckled staining pattern (46). Expression of V5-tagged UUKV NSs exhibited the characteristic punctate, cytoplasmic staining described above (Fig. 1A and D). We found that overexpression of FLAG-tagged MAVS in HEK293T cells resulted in the distinct speckled staining characteristic of the active form of MAVS (Fig. 4D). Although colocalization of UUKV NSs and MAVS was not obvious in some cells, close examination revealed partial colocalization of the UUKV NSs punctate staining with MAVS speckles (Fig. 4D). Partial colocalization of UUKV NSs with MAVS agrees with our proposed hypothesis that the interaction between MAVS and UUKV NSs is weak, which could explain its weak IFN-antagonistic activity.

### HRTV NSs antagonizes IFN induction through a direct interaction with TBK1.

**FIG 5:**
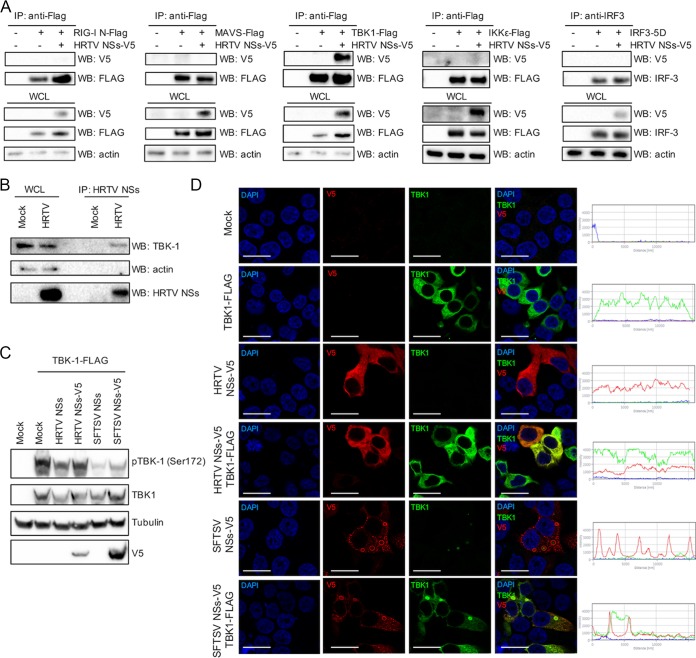
HRTV NSs interacts with TBK1. (A) HEK293T cells were cotransfected with a V5-tagged HRTV NSs-encoding plasmid along with FLAG-tagged RIG-I (N), MAVS, TBK1, or IKKε or untagged IRF3-5D-encoding plasmids. Transfected lysates were subjected to coimmunoprecipitation with beads conjugated to FLAG or IRF3 antibodies. V5-tagged HRTV NSs was detected through Western blotting with an anti-V5 antibody. (B) Immunoprecipitation of HRTV NSs in infected cells. Cell lysates of A549 cells infected with HRTV (MOI 10 FFU/cell) were subjected to immunoprecipitation with an anti-HRTV NSs antibody. Western blotting was performed on IP eluates to detect the presence of TBK1 and HRTV NSs in HRTV-infected cells. (C) HEK293T cells were cotransfected with plasmids expressing untagged or V5-tagged HRTV or SFTSV NSs and FLAG-tagged TBK1 to induce interferon induction. At 24 h posttransfection, cell lysates were harvested and utilized for Western blotting of TBK1, TBK1 phosphorylated at Ser172, tubulin, and V5 with the appropriate antibodies. WB, Western blot. (D) Indirect immunofluorescent staining of HEK293T cells transiently expressing TBK1-FLAG and V5-tagged HRTV or SFTSV NSs and probed with anti-TBK1 and anti-V5 antibodies 24 h posttransfection. Subcellular localization of NSs proteins (red), TBK1 (green), and nuclei stained with DAPI (blue) was analyzed by confocal microscopy. Intensity profile graphs are shown to the right of the image. Scale bars indicate 20 μm.

To elucidate the interacting partners of HRTV NSs, we took an approach similar to the one taken for UUKV NSs. Cotransfection of V5-tagged HRTV NSs and each of the FLAG-tagged IFN induction components resulted in the co-IP of HRTV NSs in the presence of TBK1 only (Fig. 5A). Similar results were observed for SFTSV NSs, as previously reported (Fig. S3) (30, 31). In the context of a virus infection, endogenous TBK1 was also coimmunoprecipitated with HRTV NSs (Fig. 5B). The interaction observed in transfected and infected cells also correlates with the inhibitory effect of HRTV NSs observed in our luciferase reporter assays (Fig. 3).

10.1128/mSphere.00234-17.3 FIG S3  SFTSV NSs interacts with TBK1. HEK293T cells were cotransfected with V5-tagged SFTSV NSs along with FLAG-tagged RIG-I N, MAVS, TBK1, or IKKε or untagged IRF3-5D. Cell lysates were subjected to coimmunoprecipitation with beads conjugated to FLAG or IRF3 antibodies, and V5-tagged SFTSV NSs was detected through Western blotting. Download FIG S3, TIF file, 4.8 MB. Copyright © 2017 Rezelj et al. 2017 Rezelj et al. This content is distributed under the terms of the Creative Commons Attribution 4.0 International license.

10.1128/mSphere.00234-17.4 FIG S4  Uncropped images pertaining to Fig. 2. Download FIG S4, TIF file, 12.2 MB. Copyright © 2017 Rezelj et al. 2017 Rezelj et al. This content is distributed under the terms of the Creative Commons Attribution 4.0 International license.

TBK1 activation is achieved through phosphorylation of Ser172 (pTBK1) within its classical kinase activation loop by the action of IKKβ. Furthermore, TBK1 can also autophosphorylate at Ser172, leading to its activation when overexpressed in HEK293T cells (47–50). Thus, we investigated whether HRTV or SFTSV NSs could prevent the activation of TBK1 by inhibiting its phosphorylation at Ser172, thereby blocking IFN induction. HEK293T cells were induced by a plasmid encoding FLAG-tagged TBK1 in the presence of 250 ng of untagged or V5-tagged NSs proteins and were subsequently harvested for analysis of TBK1 phosphorylation. TBK1 phosphorylated at Ser172 could be readily detected in the mock cells stimulated with TBK1 (Fig. 5C). Reduced levels of phosphorylated TBK1 were observed in the presence of HRTV or SFTSV NSs. However, the block in phosphorylation appeared to be greater in the presence of SFTSV NSs than in the presence of HRTV NSs whereas the levels of total TBK1 remained unchanged under all experimental conditions. Of note, the difference in the levels of efficiency of antagonism of TBK1 phosphorylation could once again be explained by the levels of HRTV NSs expression being lower than those of SFTSV NSs expression, as detected by Western blotting of transfected cell lysates (Fig. 5C).

We examined the subcellular localization of TBK1 in the presence or absence of HRTV or SFTSV NSs in HEK293T cells. On its own, TBK1 showed cytoplasmic staining (Fig. 5D). There was a clear sequestration of TBK1 to the inclusion bodies in SFTSV NSs-transfected cells, as previously described by several groups. However, in cells transiently expressing HRTV NSs, TBK1 remained cytoplasmic but colocalized with the HRTV NSs signal (Fig. 5D). Interestingly, despite exhibiting very different cellular localization, the strategy by which the antagonistic function of HRTV NSs occurs appears to mirror that of SFTSV, i.e., blocking the phosphorylation and hence activation of TBK1. Thus, our findings suggest that HRTV NSs and SFTSV NSs share some key characteristics: IFN antagonist activity and interaction with TBK1.

### HRTV and SFTSV, but not UUKV NSs, impair type I IFN signaling through an interaction with STAT2 but do not inhibit type II IFN signaling.

**FIG 6:**
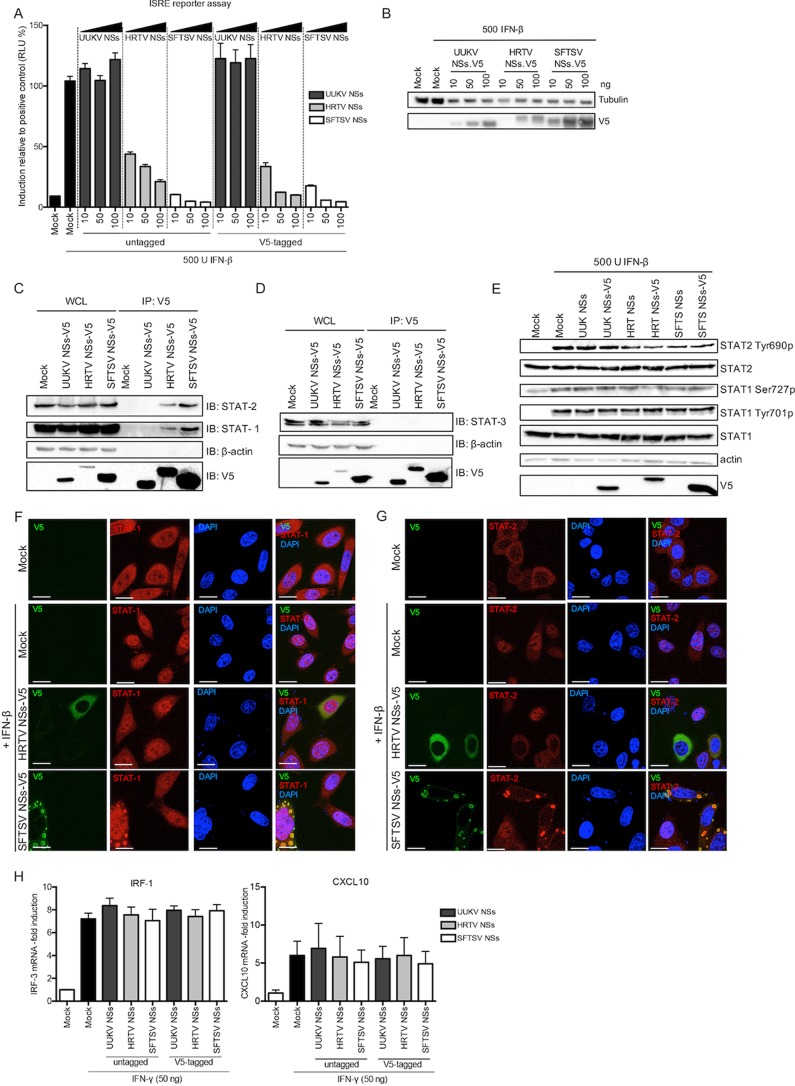
HRTV and SFTSV NSs, but not UUKV NSs, inhibits JAK/STAT IFN signaling. (A) ISRE reporter assay in the presence of tick-borne *Phlebovirus* NSs proteins. HEK293T cells were transfected with a Firefly luciferase reporter plasmid under the control of an ISRE promoter and a *Renilla* luciferase control plasmid in the presence of the indicated amounts of untagged or V5-tagged UUKV, HRTV, or SFTSV NSs proteins. The cells were stimulated with IFN-β (500 U/ml) 24 h posttransfection and lysed 18 h later for analysis. Induction was normalized against the induction control, whose results were assigned a value of 100%. The data represent results of three independent experiments performed in duplicate (*n* = 3), presented as fold induction means ± SEM. RLU, relative light units. (B) Western blots of transfected cell lysates. (C and D) Cell lysates from HEK293T cells transiently expressing V5-tagged UUKV, HRTV, or SFTSV NSs were immunoprecipitated with an anti-V5 antibody and analyzed by Western blotting with STAT1 and STAT2 antibodies (C) or with a STAT3 antibody (D). (E) Phosphorylation of STAT1 and STAT2 in HEK293T cells upon treatment with recombinant IFN-β. HEK293T cells transiently expressing untagged or V5-tagged UUKV, HRTV, or SFTSV NSs were treated with recombinant IFN-β 24 h posttransfection. Upon 30 min of IFN-β treatment, the cell lysates were harvested and utilized for Western blotting of STAT2, STAT2 phosphorylated at Tyr690, STAT1, STAT1 phosphorylated at Ser727 or Tyr701, actin, and V5 with the appropriate antibodies. (F and G) HeLa cells were transfected with plasmids encoding V5-tagged HRTV or SFTSV NSs. At 24 h posttransfection, the cells were treated with IFN-β (1,000 U/ml) for 30 min, fixed, permeabilized, and probed with (V5) STAT1 (F) and STAT2 (G) antibodies to visualize the subcellular localization of endogenous STATs (red), V5-tagged NSs (green), and DAPI-stained nuclei (blue) by confocal microscopy. Scale bars indicate 20 μm. (H) HEK293T cells transiently expressing untagged or V5-tagged UUKV, HRTV, or SFTSV NSs proteins were treated with 50 ng IFN-γ. At 24 h posttreatment, total cellular RNA was isolated and subjected to RT-qPCR to examine mRNA levels of IP-10 and CXCL10. Fold increase was derived by normalizing relative mRNA levels of the target to GAPDH mRNA levels using a ΔΔ*CT* method.

To assess whether UUKV and HRTV NSs could inhibit type I IFN signaling, we transfected HEK293T cells with a reporter plasmid encoding luciferase under the control of an interferon-stimulated response element (ISRE) promoter along with constructs expressing the viral NSs proteins, using SFTSV NSs as a control, as SFTSV NSs has been shown to act as a potent antagonist of IFN signaling (30–34). Interestingly, UUKV NSs was unable to abrogate type I IFN signaling. In comparison, HRTV NSs and SFTSV NSs exhibited a strong inhibitory effect on ISRE promoter activation (Fig. 6A). As already noted, expression of HRTV NSs was lower than that of UUKV NSs or SFTSV NSs in these assays, which was demonstrated by Western blotting of the cell lysates transfected with V5-tagged NSs proteins (Fig. 6B).

Other published data show that SFTSV NSs directly interacts with and sequesters STAT1 and STAT2 into inclusion bodies to block type I IFN signaling (33, 34). To investigate whether HRTV NSs was also capable of these interactions, we performed immunoprecipitation of transfected V5-tagged NSs proteins (Fig. 6C). In agreement with our reporter assay, no interaction between UUKV NSs and STAT1 or STAT2 was observed. Like SFTSV NSs, endogenous STAT1 and STAT2 were coimmunoprecipitated with HRTV NSs, suggesting that the IFN signaling interacting partners of these two proteins are conserved (Fig. 6C). Interestingly, STAT2 levels in IP eluates were higher than those of STAT1 (relative to the respective total expression levels in the whole-cell lysate). Indeed, a longer exposure was required to detect STAT1 in immunoprecipitated samples, suggesting that the interaction of HRTV NSs or SFTSV NSs with STAT2 is stronger than that with STAT1. It is also possible that STAT1-STAT2 heterodimers are precipitated in the presence of HRTV and SFTSV NSs proteins and therefore that the precipitated STAT1 is not a result of a direct interaction with the NSs proteins but is rather a result of an indirect interaction through STAT2.

STAT3 is a pleiotropic STAT that has been implicated in modulating inflammatory responses in type I IFN signaling (51) as well as in regulating T cell proliferation by preventing apoptosis (52) and CD4^+^ and CD8^+^ T cell survival and differentiation (53, 54). With this in mind, we also investigated whether the NSs proteins of tick-borne phleboviruses would interact with STAT3. No STAT3 was pulled down following immunoprecipitation of V5-tagged proteins (Fig. 6D).

The ultimate translocation of STAT1 and STAT2 proteins to the nucleus to activate the promoter of ISGs occurs via their phosphorylation and dimerization (55, 56). While one study has shown that only STAT2 (but not STAT1) phosphorylation is inhibited by SFTSV NSs (34), another study has shown that the phosphorylation of STAT2 as well as of STAT1 at position Ser727 is blocked by SFTSV NSs (33). We examined the ability of HRTV or SFTSV NSs to inhibit STAT1 or STAT2 phosphorylation using UUKV NSs as a negative control, as UUKV NSs did not block type I IFN signaling (Fig. 6A). HEK293T cells transiently expressing untagged or V5-tagged UUKV, HRTV, or SFTSV NSs proteins were treated with recombinant IFN-β for 30 min, and the cell lysates were harvested for Western blotting 24 h posttransfection. Unlike Chaudhary et al. (33), we observed no difference in the levels of STAT1 or phosphorylated STAT1 (at position Ser727 or position Tyr701) in the presence of SFTSV NSs compared to the mock control. Similar results were observed for UUKV and HRTV NSs proteins (Fig. 6E). While in the presence of the tick-borne *Phlebovirus* NSs proteins STAT2 levels also remained unchanged compared to those seen with the mock control, we observed a reduction in the levels of STAT2 phosphorylation (position Tyr690) in the presence of HRTV and SFTSV NSs proteins compared to the levels found in the mock control and the UUKV NSs-transfected controls (Fig. 6E).

We next sought to compare the effect of HRTV NSs on the translocation of STAT1 and STAT2 to the nucleus upon IFN stimulation to that of SFTSV NSs. To do this, we used HeLa cells, which have been utilized previously to visualize the sequestration of STAT1 and STAT2 into SFTSV NSs-formed inclusion bodies (33). HeLa cells transiently expressing V5-tagged HRTV or SFTSV NSs were treated with IFN-β for 30 min at 24 h p.t. prior to analysis with V5 and STAT1 or STAT2 antibodies. Although STAT1 was sequestered into the characteristic inclusion bodies in cells transiently expressing SFTSV NSs, its translocation to the nucleus due to IFN-β stimulation was not completely inhibited, as nuclear STAT1 could also be detected (Fig. 6F). Expression of HRTV NSs did not result in an accumulation of STAT1 into inclusion bodies as noted for SFTSV; instead, STAT1 appeared diffused throughout the cytoplasm, as well as in the nucleus (Fig. 6F). In contrast to what was observed with STAT1, no nuclear translocation of STAT2 was detected in cells transiently expressing V5-tagged HRTV or SFTSV NSs upon IFN treatment (Fig. 6G). As with TBK1, STAT2 remained diffused in the cytoplasm in the presence of HRTV NSs, in comparison to a clear sequestration into the inclusion bodies formed by SFTSV NSs. Thus, these results indicate that the interaction of HRTV NSs or SFTSV NSs protein with STAT2 (but not STAT1) results in the efficient inhibition of IFN-β-induced STAT2 nuclear translocation. Perhaps the leakage of STAT1 into the nucleus in the presence of HRTV NSs or SFTSV NSs following IFN-β treatment is a result of a weak (or indirect) interaction between the NSs proteins and STAT1, as noted from our co-IP experiments.

As type II IFN signaling is mediated by STAT1 homodimers, we investigated whether the weak or indirect interaction of HRTV and SFTSV NSs with STAT1 could result in an inhibitory effect upon type II IFN signaling. mRNA levels of two IFN-γ-induced ISGs (IRF-1 and CXCL10) were investigated following IFN-γ treatment of HEK293T cells transiently expressing HRTV or SFTSV NSs proteins, using UUKV NSs as a control (Fig. 6H). No effect was observed on mRNA levels of IRF-1 and CXCL10 in cells transiently expressing UUKV, HRTV, or SFTSV NSs proteins following IFN-γ treatment. This finding suggests that the interaction of HRTV and SFTSV NSs with STAT1 does not inhibit type II IFN signaling.

To summarize, our results indicate that impairment of type I IFN signaling by HRTV and SFTSV NSs occurs mainly in a STAT2-dependent manner, whereas no impairment of IFN signaling by UUKV NSs was detected. Additionally, only the phosphorylation and nuclear translocation of STAT2 are efficiently impaired by HRTV and SFTSV NSs proteins upon IFN-β treatment. These observations suggest that both HRTV and SFTSV NSs proteins have a conserved direct interaction with STAT2 which enables the proteins to efficiently antagonize type I IFN signaling (Fig. 6A). On the other hand, their weak or indirect interaction with STAT1 does not enable HRTV and SFTSV NSs proteins to inhibit type II IFN signaling (Fig. 6H).

### Comparison of the levels of TiBo *Phlebovirus* sensitivity to the interferon response.

**FIG 7:**
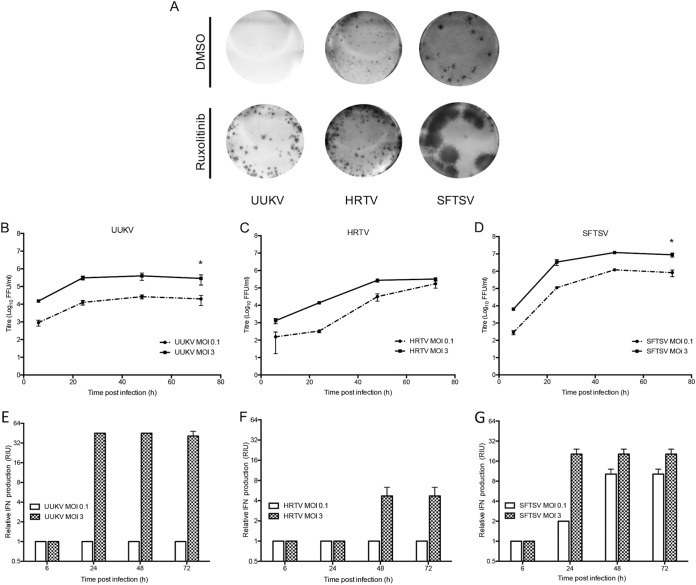
Sensitivity of tick-borne phleboviruses to IFN. (A) Focus-forming assays of UUKV, HRTV, and SFTSV on A549 cells 6 days p.i. The overlay was supplemented with JAK/STAT inhibitor ruxolitinib (0.4 μM) or the equivalent volume of the vehicle control DMSO. Foci were detected using anti-UUKV, HRTV, or SFTSV N antibodies. (B to D) Growth curves of UUKV (B), HRTV (C), or SFTSV (D) at a low MOI (0.1 FFU/cell) and a high MOI (3 FFU/cell) in A549 cells. The cell culture supernatant of triplicate wells was collected at the indicated time points, and virus titers were determined through focus-forming assays. *, *P* ≤ 0.05. (E to G) Samples from the experiments described for panels B to D were subjected to UV inactivation, and the relative IFN units were quantified through a biological IFN assay. Error bars indicate SD of the means. The results shown in this figure are representative of 2 experimental repeats.

Different strains of mosquito-borne Rift Valley fever virus (RVFV) exhibit differential sensitivities to the action of IFN depending on the capacity of the NSs proteins of the various strains to efficiently block the type I IFN response (57). To investigate the sensitivity of tick-borne phleboviruses to virus-induced IFN, we examined the effect of a JAK1/2 inhibitor, ruxolitinib (previously described in reference 58), on focus formation. While UUKV foci were undetectable in A549 cells, treatment with ruxolitinib restored the ability of UUKV to form foci in these cells (Fig. 7A). In contrast, ruxolitinib treatment had no significant effect on HRTV focus size. Unexpectedly, and even though the SFTSV genome encodes an NSs protein that is a potent inhibitor of the IFN system (30–34), we observed a significant increase in SFTSV focus size in A549 cells treated with ruxolitinib (Fig. 7A). Thus, despite encoding a potent IFN antagonist, SFTSV infection still results in IFN induction (Fig. 7G), thereby limiting virus infection in neighboring cells. To ensure that this effect was not due to the presence of defective interfering particles in our SFTSV stock, the virus was subjected to plaque purification. Four different newly plaque-purified SFTSV stocks yielded results similar to those obtained with our laboratory SFTSV stock (data not shown).

To corroborate the results obtained with ruxolitinib treatment, the growth kinetics of UUKV, HRTV, and SFTSV were compared at a low MOI and a high MOI (0.1 FFU/cell and 3 FFU/cell, respectively) in A549 cells. At the indicated time points, cell culture supernatants were harvested, virus titers were determined, and the relative amounts of IFN produced were quantified through biological IFN assays. The findings presented in Fig. 7B to G support the results obtained by measuring IFN sensitivity using ruxolitinib treatment. At the low MOI, infection with UUKV and SFTSV resulted in mean peak titers of 2 × 10^4^ FFU/ml and 8.2 × 10^5^ FFU/ml at 72 h p.i., respectively, approximately 1-log lower than the titers seen when cells were infected at the high MOI (Fig. 7B and D). While UUKV infection induced IFN by 24 h p.i. following the high-MOI infection, no IFN could be detected at the low MOI (Fig. 7E). The lack of IFN production by UUKV at the low MOI could be explained by the low replication levels seen throughout the time course, as only a 1-log increase in virus titer was observed by 72 h p.i. On the other hand, faster growth kinetics exhibited by SFTSV (a 3-log increase in virus titer) resulted in increased induction of IFN by SFTSV at the low MOI (Fig. 7G). In contrast to UUKV and SFTSV, the high and low MOI growth curves of HRTV showed similar mean titers by 72 h p.i. (at the low MOI, 1.7 × 10^5^ FFU/ml; at the high MOI, 3.3 × 10^5^ FFU/ml) (Fig. 7D). Furthermore, even at the high MOI, HRTV infection resulted in very little induction of IFN (4 relative IFN units [RIU]) by 72 h p.i. (Fig. 7F). Though surprising, our data showing the induction of IFN by SFTSV despite its being equipped with a potent antagonist of the IFN system agree with a recent study. That study demonstrated that sera from SFTSV-infected patients showed high concentrations of IFN-α compared to sera from healthy patients and that the concentration of IFN-α in patients with severe SFTSV was significantly higher than that in patients with mild SFTS (59).

Taken together, these data demonstrate that UUKV and SFTSV cannot efficiently antagonize IFN production during infection and that induction of IFN leads to reduced spread of the virus to neighboring cells. On the other hand, we show that HRTV can limit IFN production following infection.

## DISCUSSION

Phleboviruses have a trisegmented negative or ambisense RNA genome that can activate IFN induction by the stimulation of RIG-I. Induction of type I IFN at an early stage following virus infection can have protective effects against virus infection, which is best illustrated by the high susceptibility of type I IFN receptor knockout mice to virus infection compared to wild-type mice (57, 60, 61). Consequently, phleboviruses require efficient mechanisms to antagonize the IFN response. Recent reviews have highlighted the importance of elucidating the countermeasures employed by phleboviruses to hinder the IFN response (62, 63). These recommendations come with the realization that, until the recent emergence of highly pathogenic SFTSV, most studies focused on the ability of the NSs protein of mosquito-borne phleboviruses, in particular, RVFV NSs protein, to overcome the IFN system. However, unlike most other *Phlebovirus* NSs proteins, RVFV NSs localizes to the nucleus, forming unique filamentous structures in the nuclei of infected cells (64–68). Part of the IFN-antagonistic activity of RVFV NSs is attributed to its nuclear localization, which allows it to suppress host cell transcription generally but also IFN-β mRNA synthesis specifically (69–71). RVFV NSs has also been implicated in preventing the inhibition of translation by the proteasome-dependent downregulation of double-stranded RNA-dependent protein kinase (PKR) (72–76). Like RVFV, the NSs protein of mosquito-borne Toscana virus (TOSV) can induce proteasome-mediated degradation of PKR (77), but it can additionally inhibit the production of IFN through a direct interaction with RIG-I (78, 79). The NSs protein of Punta Toro virus (PTV; also mosquito-borne) acts as a suppressor of the IFN response by inhibiting host cell transcription like RVFV NSs but does not affect PKR levels (77, 80).

With the emergence of SFTSV in China and the discovery of HRTV in the United States, there is now a pressing need to understand the molecular mechanisms of virulence of these pathogens and those of other previously described and neglected tick-borne phleboviruses. The discovery that SFTSV NSs forms unique cytoplasmic inclusion bodies which act to sequester elements from the IFN induction and IFN signaling pathways suggested that *Phlebovirus* NSs proteins have evolved highly divergent mechanisms to counteract the human IFN response (30–34, 81). This is underscored by the fact that within the *Phlebovirus* genus, the NSs protein sequence has extremely low conservation at the amino acid level in comparison to the other viral proteins (5).

**FIG 8:**
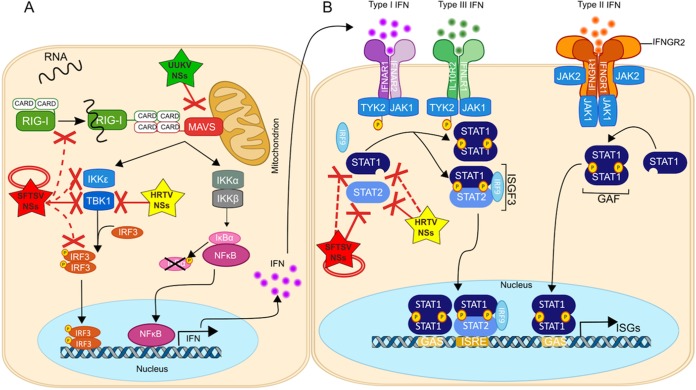
Schematic summary of the mechanisms by which tick-borne *Phlebovirus* NSs proteins inhibit the canonical IFN induction and signaling pathways. Following bunyavirus infection, the generation of ssRNA with uncapped 5′ triphosphate ends during uncoating, transcription, or replication results in ssRNA binding to the RNA helicase RIG-I. RIG-I is activated in turn, and exposure of its two associated CARD domains allows the recruitment of the adaptor MAVS through two CARD-CARD interactions. Activation of MAVS leads to the subsequent activation of kinases such as TBK1 and/or IKKε, which leads to concomitant phosphorylation of IRF3 at specific serine residues. (A) Phosphorylation of IRF3 leads to its dimerization and translocation to the nucleus, where IRF3 dimers ultimately stimulate transcription of genes under the control of the IFN-α/β promoter, resulting in the production of IFN-α/β. (B) Signal transduction of type I IFNs initiates through the binding of secreted IFN-α/β to type I IFN receptors (a heterodimer of IFNAR1 and IFNAR2) and the activation of multiple downstream signaling pathways. Signal transduction of type I, II, and III IFN initiates through the binding of secreted IFN to the respective IFN receptors and the activation of multiple downstream signaling pathways. Type I and type III IFN signaling pathways are mainly mediated via STAT1-STAT2 heterodimers. Receptor-associated kinases JAK1 and TYK2 become activated and phosphorylate STAT1 and STAT2, respectively. Phosphorylated STATs can heterodimerize and recruit IRF9 for the assembly of the heterotrimer complex ISGF3. ISGF3 translocates to the nucleus and binds to ISRE, leading to the induction of many IFN-stimulated genes (ISG). Type III IFN signaling is more commonly associated with signaling by homodimerization of STAT1, which can translocate to the nucleus and activate GAS promoters, also initiating ISG transcription. Tick-borne *Phlebovirus* NSs proteins have evolved several countermeasures to block the IFN pathway. SFTSV NSs (red) directly interacts with and sequesters TBK1 and STAT2 into inclusion bodies to spatially isolate these elements. Additionally, through its interaction with TBK1 and STAT2, SFTSV NSs can also indirectly sequester IKKε, IRF-3, and STAT1 into the inclusion bodies. An interaction between SFTSV NSs and TRIM-25 also facilitates the spatial isolation of RIG-I, in an indirect manner. HRTV NSs (yellow) can also block IFN induction through a direct interaction with TBK1 and block IFN signaling by a direct interaction with STAT2. However, as HRTV NSs does not form inclusion bodies as SFTSV NSs does, its mechanism of antagonism is different from that of SFTSV NSs. UUKV NSs (green) blocks IFN induction only, targeting MAVS. For references, see the text. Dashed red lines indicate indirect interactions. Solid red lines indicate direct interactions. For references, see the text. CARD, caspase recruitment domain; GAS, gamma-activated sequence; IFNAR, interferon-α/β receptor; IFNGR1, interferon gamma receptor 1; IκB, inhibitor of kappa B; IKK, inhibitor of nuclear factor kappa-B kinase subunit alpha; IKK β, inhibitor of nuclear factor kappa-B kinase subunit beta; IKKε, IκB kinase-ε; IRF, IFN-regulatory factor; ISGF3, IFN-stimulated gene factor 3; ISGs, IFN-stimulated genes; ISRE, IFN-stimulated response elements; JAK1, Janus kinase 1; MAVS, mitochondrial antiviral signalling protein; NFκB, nuclear factor kappa-light-chain enhancer of activated B cells; RIG-I, retinoic acid-inducible gene I; STAT, signal transducer and activator of transcription; TBK1, TANK-binding kinase 1; TYK2, tyrosine kinase 2.

In this report, we highlight the diverse mechanisms that tick-borne *Phlebovirus* NSs proteins employ to antagonize the IFN response, by demonstrating that UUKV NSs and HRTV NSs utilize strategies different from those employed by the well-studied SFTSV NSs to subvert this powerful antiviral response. The proposed model of tick-borne *Phlebovirus* NSs antagonism of the IFN response is summarized in Fig. 8. Our reporter assays showed that the NSs protein of apathogenic UUKV acts as a weak antagonist of IFN induction, but not of IFN signaling, which agrees with our previous studies (35) (Fig. 2 and 6). This is in comparison to the strong inhibition of IFN-β and ISRE promoter activation seen with the NSs proteins belonging to the more pathogenic HRTV and SFTSV. As UUKV has been associated with infection in birds (14, 82, 83), it would be of interest to investigate in future studies whether its NSs protein has adapted to antagonize the avian innate immune system with higher efficiency than was seen with the human innate immune system tested here. Our reporter assays and coimmunoprecipitation studies further demonstrated that the molecular mechanism regulating the inhibition of IFN induction by UUKV NSs likely operates through a direct interaction with MAVS, an effector protein involved in the early stages of the IFN induction pathway (Fig. 3 and 4). Examination of this interaction through confocal microscopy studies revealed that some punctate cytoplasmic structures of UUKV NSs colocalize with speckle-like structures formed by MAVS (Fig. 4D), which may inhibit MAVS activation and downstream signaling of IFN induction. Toscana virus (TOSV) NSs protein has also been shown to inhibit the induction of type I IFN at early stages of the IFN induction pathway by targeting RIG-I for proteasomal degradation (78). The influenza A virus PB1-F2 protein has also been shown to suppress IFN induction by binding to MAVS and altering the mitochondrial membrane potential (84, 85), which is required for MAVS-mediated IFN induction (86). Studies are in progress to understand whether UUKV NSs utilizes mechanisms to suppress IFN induction that are similar to those described for influenza virus PB1-F2 protein. As it stands, and to our knowledge, targeting MAVS to antagonize the type I IFN response is a novel strategy described for *Phlebovirus* NSs proteins.

Our results show, for the first time, that HRTV NSs acts as a potent antagonist of both IFN induction and type I IFN signaling (Fig. 2 and 6). Reporter assays and co-IP experiments enabled us to determine that the ability of HRTV NSs to block IFN induction and type I IFN signaling is facilitated through a direct interaction with TBK1 and STAT2, respectively (Fig. 5 and 6). Despite HRTV NSs sharing only 63% amino acid identity with SFTSV NSs, the interactions reported here are conserved with those of SFTSV NSs, which also interacts with TBK1 and STAT2, antagonizing their phosphorylation to inhibit the IFN response (30, 31, 34). However, while SFTSV NSs spatially isolates TBK1 and STAT2 into inclusion bodies, the interactions between HRTV NSs and TBK1 and STAT2 were notably different in terms of subcellular localization, as these proteins remained diffused in the cytoplasm in the presence of HRTV NSs (Fig. 5 and 6). It was previously reported that the N-terminal PxxP motif (amino acid residues 66 to 69) of SFTSV NSs is required for the formation of inclusion bodies (31). Simultaneous substitution of Pro66 and Pro69 resulted in diffused cytoplasmic localization and affected the IFN-antagonistic activity of SFTSV NSs. Surprisingly, the PxxP motif was conserved in HRTV NSs (see Fig. S1 in the supplemental material), indicating that the motif may be required but is not sufficient for inclusion body formation and the IFN antagonistic activity of NSs. Nonetheless, a direct interaction of HRTV NSs with TBK1 in the cytoplasm, rather than sequestration to inclusion bodies, was sufficient to prevent its activation by blocking TBK1 phosphorylation of Ser172 and the subsequent phosphorylation of downstream factors.

In terms of IFN signaling, our co-IP assays demonstrated that HRTV and SFTSV NSs could interact with both STAT1 and STAT2 (Fig. 6C). However, a stronger interaction was observed with STAT2 than with STAT1. Additionally, IFN-β-induced phosphorylation and nuclear translocation of STAT2, but not STAT1, were efficiently inhibited in the presence of HRTV or SFTSV NSs proteins (Fig. 6E to G).

In a manner similar to its interaction with TBK1, the mechanism by which SFTSV NSs inhibits STAT1 and STAT2 translocation to the nucleus operates through their spatial isolation in inclusion bodies (34). No spatial isolation of STAT1 or STAT2 was observed in the presence of HRTV NSs, suggesting simply that an interaction between HRTV NSs and STAT2 may block phosphorylation of STAT2 and its heterodimerization with STAT1, consequently inhibiting the translocation of STAT1-2 heterodimers to the nucleus and thus inhibiting type I and type III IFN signaling. It remains to be elucidated whether the coimmunoprecipitation of STAT1 in the presence of HRTV and SFTSV NSs is due to an indirect interaction through the precipitation of STAT1-2 heterodimers or simply to a weak interaction. As it stands, the weak interaction or lack of direct interaction with STAT1 may play a role in the differential regulation of type I and type II IFN signaling by HRTV and SFTSV NSs. In canonical type I IFN signaling, STAT1-STAT2 heterodimers, in association with IRF-9, translocate to the nucleus and bind to ISREs. Under conditions of activation through type I or type II IFN, STAT1 can also form homodimers, which translocate to the nucleus to activate gamma-activated site (GAS) elements. Activation of GAS elements by STAT1 homodimers results in the production of proinflammatory cytokines, such as tumor necrosis factor (TNF), interleukin-6 (IL-6), and P40, which can cause severe disease (87). Our results indicate that HRTV and SFTSV NSs proteins are unable to inhibit type II IFN signaling (Fig. 6H), an observation which is supported by our data showing a weak interaction or a potential lack of direct interaction between these proteins and STAT1 as well as the inability of HRTV and SFTSV NSs proteins to block the nuclear translocation of STAT1. Furthermore, STAT3, which has also been implicated in modulating inflammatory responses (88), did not act as an interacting partner for UUKV, HRTV, or SFTSV NSs. Therefore, our results indicate that HRTV NSs can suppress type I IFN signaling through a direct interaction with STAT2 in a manner different from that seen in the sequestration of STAT2 into SFTSV NSs inclusion bodies. The weak interaction or lack of direct interaction of HRTV and SFTSV NSs with STAT1, and the lack of an interaction with STAT3, could act as an explanation for the strong cytokine-mediated inflammatory responses suggested to be responsible for disease progression in SFTSV mouse models as well as in patients diagnosed with SFTS (89, 90) and for the symptoms (identical to those seen in SFTS disease) that have been observed in HRTV patients (9, 10).

The first indication that the NSs protein of a *Phlebovirus* had an IFN antagonist role was the finding that, in comparison to virulent RVFV strain ZH548, RVFV strains bearing mutations in the NSs gene (MP12 and clone 13) induced IFN and were virulent in mice lacking the type I IFN receptor but not in wild-type mice (57). Thus, given that the sensitivity of RVFV to IFN is dictated by the capacity of the virus NSs protein to efficiently block the production of IFN following infection, we aimed to investigate the sensitivity to IFN of the tick-borne phleboviruses used in this report. Our results show that in IFN-competent A549 cells, the foci produced by UUKV were undetectable and the foci produced by SFTSV were small (Fig. 7A). However, when IFN signaling was impaired by addition of the JAK1/2 inhibitor ruxolitinib to the focus-forming assay overlay, UUKV foci could be detected and SFTSV formed significantly larger foci. These data suggest that the IFN response mounted as a result of virus infection results in an antiviral state in neighboring infected cells, which limits UUKV and SFTSV spread. Similarly, the inability of UUKV and SFTSV to reach similar peak titers during infections at low MOI and high MOI could be explained by the sensitivity of the viruses to induced IFN, limiting virus replication. Notably, some differences were observed in the ability of the viruses to interact with cells in an IFN-induced state. UUKV exhibited poor replication kinetics, presumably due to its inability to cope with the action of virus infection-induced IFN (Fig. 7B and E). These results are in line with our previous finding that UUKV NSs is a weak IFN antagonist (35), as the inability of UUKV to efficiently circumvent the IFN response may be a result of the weak IFN-antagonistic activity of its NSs protein. Comparatively, SFTSV exhibited faster replication kinetics throughout the time course despite inducing more IFN, suggesting that SFTSV is less sensitive to the action of IFN than UUKV (Fig. 7B, D, E, and G).

Other studies and the results described here suggested that the NSs protein of SFTSV is a potent IFN antagonist. However, a recent report showed that sera from SFTSV-infected patients show high concentrations of IFN-α compared to sera from healthy patients (59). Our results explain these observations and indicate that, despite encoding a potent IFN antagonist (Fig. 2 and 6), in the context of a virus infection, the amount of IFN induced during SFTSV infection is not completely overcome by the action of its viral IFN antagonist (Fig. 7). Perhaps SFTSV has evolved such a potent IFN antagonist to compensate for the generation of a large number of PAMPs during virus replication that result in the rapid induction of the IFN response—an interesting hypothesis and avenue for future studies. Interestingly, and in comparison to SFTSV, no significant difference was observed in HRTV focus size in A549 cells in comparisons of functional and nonfunctional IFN signaling responses (Fig. 7A). Low- and high-MOI HRTV infections resulted in similar peak titers, and little IFN was detected, suggesting that the virus can efficiently control IFN production following infection, which the data presented in the manuscript suggest is mediated by its NSs protein.

To conclude, our findings serve to expand our knowledge of the differential strategies evolved by phleboviruses to modulate host innate immune responses and highlight that, despite being poorly conserved in terms of amino acid sequence, the NSs proteins of phleboviruses retain their IFN antagonistic function.

## MATERIALS AND METHODS

### Cells and viruses.

A549, HeLa, and Vero E6 cells (commonly used cell lines originally obtained from the European Collection of Authenticated Cell Cultures [ECACC] and previously described in references 64 and 91; Vero E6 cells were from Institut Pasteur) were grown in Dulbecco’s modified Eagle’s medium (DMEM) supplemented with 10% fetal calf serum (FCS). HEK293T cells (ECACC) were grown in DMEM supplemented with 10% FCS and 0.1 mM MEM nonessential amino acids (NEAA). BSR cells (a variant of BHK-21 cells; kindly provided by Karl-Claus Conzelmann) were grown in Glasgow's MEM (GMEM) supplemented with 10% tryptose phosphate broth (TPB) and 10% FCS. Cells were maintained at 37°C with 5% CO_2_.

The wild-type UUKV strain used in this study was derived from the prototype S-23 strain and grown in BSR cells as previously described (35, 92). HRTV (isolated from patient 2 as described in reference 9) was a kind gift by R. Tesh (World Reference Centre for Emerging Viruses and Arboviruses, Galveston, TX). The SFTSV strain used was a plaque-purified cell culture-adapted stock strain called Hubei 29pp (HB29pp) provided by A. Lambert (CDC Arbovirus Diseases Branch, Division of Vector-Borne Infectious Diseases, Fort Collins, CO) (93). Working stocks of HRTV and SFTSV were generated in Vero E6 cells by infection at a low multiplicity of infection (MOI) and by harvesting the cell culture medium 7 days p.i. All experiments performed with HRTV or SFTSV were conducted under containment level 3 (CL-3) conditions approved by the UK Health & Safety Executive.

### Virus titration by plaque- or focus-forming assays.

Virus titers were determined by focus-forming assays in BSR cells for UUKV and by plaque assay in Vero E6 cells for HRTV and SFTSV. Briefly, confluent monolayers of cells were infected with serial dilutions of virus made in phosphate-buffered saline (PBS) containing 2% FCS and incubated for 1 h at 37°C, followed by the addition of a GMEM overlay supplemented with 2% FCS and 0.6% Avicel (FMC Biopolymer). The cells were incubated for 6 days before fixation and staining with crystal violet was performed to visualize HRTV and SFTSV plaques or using focus-forming assays for UUKV as described previously (35).

To investigate the effect of the JAK1/2 inhibitor ruxolitinib (Selleck Chemicals) on focus formation, stocks were prepared at 10 mM in dimethyl sulfoxide (DMSO). Following virus infection of A549 cells, the Avicel overlay was supplemented with 0.4 μM ruxolitinib or the equivalent volume of the vehicle control DMSO. Foci were detected using anti-UUKV, anti-HRTV, and anti-SFTSV nucleocapsid antibodies (58, 93, 94).

### Indirect immunofluorescence staining.

For studies involving the subcellular localization of NSs proteins, HEK293T or A549 cells were grown to subconfluence on glass coverslips (13-mm diameter) and infected at a high MOI (3 FFU or PFU/ml) with UUKV, HRTV, or SFTSV. At 24 h p.i., the cells were fixed with 4% formaldehyde–PBS. Following permeabilization with 0.1% Triton X-100–50 mM glycine–PBS, proteins were detected using rabbit anti-UUKV NSs (kindly provided by Anna Överby, Umeå University, Sweden), rabbit anti-HRTV NSs (described below), rabbit anti-SFTSV NSs (93), and secondary anti-rabbit Alexa Fluor 488 (Thermo Fisher). For indirect immunofluorescence staining of proteins following transfections, HEK293T or HeLa cells seeded in poly-l-lysine-coated or uncoated glass coverslips were transfected with the appropriate expression plasmids and fixed 24 h posttransfection. Cells were permeabilized and probed with primary antibodies mouse anti-V5 antibody (kindly provided by R. E. Randall, University of St. Andrews), rabbit anti-FLAG antibody (F7425; Sigma), rabbit anti-TBK1/NAK antibody (3013; Cell Signalling), rabbit anti-STAT1 p84/p91 antibody (sc-346; Santa Cruz Biotechnologies), and rabbit anti-STAT2 antibody (sc-476; Santa Cruz Biotechnologies) and secondary antibodies anti-rabbit Alexa Fluor 488 and anti-mouse Alexa Fluor 568 (Thermo Fisher). The coverslips were mounted on slides using Fluoromount-G with DAPI (4′,6-diamidino-2-phenylindole) (EBioscience). Fluorescently labeled proteins were visualized using a Zeiss LSM-710 confocal microscope.

### Expression plasmids and cloning.

Firefly luciferase reporter plasmids used to investigate IFN-β promoter or ISRE promoter activation, p(125)luc and p(9–27)4tkΔ(−39)lucter, have been described previously (95, 96). Control plasmid phRL-CMV coding for *Renilla* luciferase under the control of a cytomegalovirus (CMV) promoter was purchased from Promega. Untagged or C-terminally V5-tagged UUKV, HRTV, and SFTSV NSs sequences were cloned into the pCMV mammalian expression plasmid using restriction-free cloning. FLAG-tagged RIG-I N, MAVS, TBK1, IKKε, and IRF3-5D expression plasmids were kindly provided by Mirko Schmolke (University of Geneva) and have been described elsewhere (97, 98).

### Cloning of HRTV N and NSs protein into a bacterial expression plasmid.

The coding region for HRTV N protein was amplified and cloned into a modified pDEST14 vector (Invitrogen) using SacI and XhoI restriction sites, generating plasmid p14 HRTV N. Plasmid pHaloHRTV NSs131-299 that expresses a Halo fusion C-terminal domain (residues 131 to 299) of HRTV NSs protein was derived from pH6HTNHisHaloTagT7-HRTV NSs, in which the full-length HRTV NSs was cloned into the modified pH6HTNHisHaloTagT7 vector (Promega). Both p14HRTV N and pHaloHRTV NSs131-299 contain an N-terminal 6-His tag for purification of soluble HRTV N and NSs.

### Expression and purification of HRTV N and NSs protein for antibody production.

N and NSs proteins were expressed in *Escherichia coli* BL21 Rosetta2 (Merck) and C43(DE3) (a gift from Huanting Liu, University of St. Andrews; originally from Lucigen Corporation) under conditions of IPTG (isopropyl-β-d-thiogalactopyranoside) induction at 18 to 20°C for 18 h. Recombinant N and NSs proteins were purified with nickel-nitrilotriacetic acid (Ni-NTA) resin. Recombinant NSs was further purified by gel filtration chromatography using a Superdex 200 10/300-Gl column. The purified N and NSs proteins were confirmed by mass spectrometry and used for generating rabbit polyclonal antibodies (Eurogentec).

### Reporter assays.

ISRE reporter assays were carried out as previously described (85). IFN-β reporter assays were performed by cotransfection of NSs proteins with expression plasmids encoding various stimuli of the IFN-β promoter (RIG-I N, MAVS, TBK1, IKKε, and IRF3-5D) as described previously (85). Briefly, subconfluent HEK293T cells in a 24-well plate were transfected using 1.5 μl TransIT LT-1 (Mirus Bio LLC) and plasmid DNA and were lysed 24 h later. The total amount of plasmid DNA was kept constant by addition of empty control plasmid. For ISRE reporter assays, transfected cells were stimulated with 500 U/ml of universal type I IFN (PBL Assay Science) and lysed 18 h post-IFN treatment. Firefly and *Renilla* luciferase activities in the reporter assays were measured using a dual-luciferase assay kit (Promega) according to the manufacturer’s instructions.

The IFN response was also measured through biological IFN assays, as described previously (35, 64, 99). For infection experiments, IFN-competent A549 cells were infected with wt UUKV, HRTV, or SFTSV at a low (0.1 FFU/cell) or high (3 FFU/cell) MOI. The cell culture medium was collected at the indicated time points and inactivated by UV light exposure (8 W; 254 nm at a distance of 2 cm for 4 min with occasional shaking). For transfection experiments, HEK293T cells induced by transfection of an expression plasmid encoding the N terminus of RIG-I were cotransfected with the different NSs proteins, and the cell culture medium was harvested 24 h p.t. Twofold dilutions of cell culture medium or UV-inactivated medium were used to pretreat A549/BVDV-Npro cells for 24 h, followed by the addition of IFN-sensitive encephalomyocarditis virus (EMCV) (0.03 PFU/cell). Cell monolayers were stained with crystal violet 4 days later, and the relative IFN units (RIU) were calculated as 2*^N^*, where *N* is the number of two-fold dilutions giving A549/BVDV-Npro cells protection.

### Western blotting.

Western blotting was performed as previously described (35) using the following antibodies: anti-UUKV N monoclonal 8B11A3 (94), rabbit polyclonal UUKV NSs, HRTV and SFTSV polyclonal anti-N and anti-NSs (93), mouse anti-V5, mouse anti-FLAG M2 (F1804; Sigma), mouse anti-α-tubulin (T5168; Sigma), mouse anti-β-actin (A5441; Sigma), anti-TBK1/NAK (3013; Cell Signalling), rabbit anti-phospho-TBK1/NAK (Ser172) (5483S; Cell Signalling), rabbit anti-RIG-I (AT111; Enzo Life Sciences), rabbit anti-IRF-3 (FL425; Sigma), rabbit anti-IRF-3 (phospho-S386) (76493; Abcam, Inc.), rabbit anti-STAT1 p84/p91 (sc-346; Santa Cruz Biotechnologies), rabbit anti-STAT2 (sc-476; Santa Cruz Biotechnologies), rabbit antistat 1 Tyr701P (9167; Cell Signalling), rabbit antistat 1 Ser727P (9177; Cell Signalling), rabbit antistat 2 Tyr690P (4441; Cell Signalling), and rabbit anti-STAT3 (sc-482; Santa Cruz Biotechnologies).

For detection of dimerized IRF-3, cells were lysed in a nonreducing lysis buffer (50 mM Tris HCl [pH 7.5], 150 mM NaCl, 1 mM EDTA, 1% Nonidet P-40) supplemented with protease and phosphatase inhibitors, separated on a nonreducing gel, and transferred to a Hybond ECL nitrocellulose membrane (GE Healthcare Life Sciences) prior to blocking in Tris-buffered saline (TBS)–0.1% Tween 20–3% bovine serum albumin (BSA; Sigma) and probing with IRF-3 antibody. Upon incubation with horseradish peroxidase (HRP)-coupled secondary antibody, proteins were detected using Clarity ECL blotting substrate (Bio-Rad) and visualized with a Bio-Rad ChemiDoc imager.

### Coimmunoprecipitation studies.

Coimmunoprecipitation (co-IP) was carried out using either transfected or infected cell monolayers. For transfections, subconfluent HEK293T cells were cotransfected with expression plasmids encoding V5-tagged NSs proteins and plasmids encoding FLAG-tagged RIG-I N, MAVS, TBK1, and IKKε or IRF3-5D. For co-IP of NSs from virus-infected cells, HEK293T or A549 cells were infected at an MOI of 20 or 5 with UUKV or HRTV, respectively. At the indicated time points, cells were lysed in co-IP buffer (50 mM Tris [pH 7.5], 150 mM NaCl, 1 mM EDTA, 10% glycerol, 1% NP-40, supplemented with a cocktail of cOmplete protease inhibitors [Roche]) and incubated by rotation at 4°C for 30 min. UUKV-infected cells were lysed in a different co-IP buffer (25 mM HEPES [pH 7.5], 150 mM NaCl, 50 mM MgCl_2_, 1% Triton X-100, supplemented with a cocktail of cOmplete protease inhibitors [Roche]). Cell lysates were clarified by centrifugation at 12,000 rpm for 20 min at 4°C. At this stage, the whole-cell lysate (WCL) fraction was taken.

For co-IP of FLAG-tagged proteins, anti-FLAG M2 magnetic beads (Sigma) were used for incubation with clarified cell lysates at 4°C for 4 h. For co-IP of UUKV NSs or HRTV NSs, clarified cell lysates were incubated overnight at 4°C with rabbit polyclonal anti-UUKV or anti-HRTV NSs antibody, respectively, followed by the addition of protein A magnetic Dynabeads (Thermo Fisher). For co-IP of V5-tagged proteins, clarified cell lysates were incubated overnight at 4°C with mouse anti-V5 antibody, followed by the addition of protein G magnetic Dynabeads (Thermo Fisher). All beads were used according to the manufacturer’s instructions. Following incubation of the cell lysates with the beads for 1.5 h at 4°C, the beads were washed five times with co-IP buffer and two times with PBS before elution of antibody complexes was performed. Elution of proteins was carried out by the addition of reducing Laemmli buffer and boiling at 95°C for 10 min. Eluates were analyzed by Western blotting, using a VeriBlot IP secondary antibody (ab131366; Abcam, Inc.).

### Quantitative RT-PCR.

Total cellular RNA was extracted with RNAiso Plus (TaKaRa). Random hexamer primers were used to synthesize cDNA using a Transcriptor First Strand cDNA synthesis kit (Roche), and SYBR Premix *Ex Taq* II mix (TaKaRa) was used for real-time PCR (RT-PCR). Reactions were carried out in triplicate on an ABI StepOnePlus system (Applied Biosystems). The relative levels of expression of mRNA were calculated using threshold cycle (ΔΔ*C*_*T*_) analysis, and the values were normalized to the relative mRNA expression level of glyceraldehyde-3-phosphate dehydrogenase (GAPDH). The following primers were used: 5′-CCATTCTGATTTGCTGCCTTAT-3′ and 5′-TTTCCTTGCTAACTGCTTTCAGTA-3′ for CXCL10, 5′-CTGTGCGAGTGTACCGGATG-3′ and 5′-ATCCCCACATGACTTCCTCTT-3′ for IRF1, and 5′-GGAGCGAGATCCCTCCAAAAT-3′ and 5′-GGCTGTTGTCATACTTCTCATGG-3′ for GAPDH.

### Statistical analysis.

All data were analyzed using Prism 5 software (GraphPad) and are presented as means ± standard deviations (SD) or standard errors of the means (SEM). Statistical significance for the comparison of means between groups was determined by one-way or two-way analysis of variance (ANOVA) followed by *post hoc* tests. *P* values of ≤0.05 were considered significant (****, *P* ≤ 0.0001; ***, *P* ≤ 0.001; **, *P* ≤ 0.01; *, *P* ≤ 0.05).

## References

[1] Plyusnin A, Elliott RM 2011 Bunyaviridae: molecular and cellular biology. Caister Academic Press, Norfolk, United Kingdom.

[2] Elliott RM, Schmaljohn CS 2013 Bunyaviridae, 6th ed. Wolters Kluwer, Philadelphia, PA.

[3] Elliott RM, Brennan B 2014 Emerging phleboviruses. Curr Opin Virol 5:50–57. doi:10.1016/j.coviro.2014.01.011. 24607799 PMC4031632

[4] Bouloy M 2011, Molecular biology of phleboviruses. Caister Academic Press, Norfolk, United Kingdom.

[5] Yu XJ, Liang MF, Zhang SY, Liu Y, Li JD, Sun YL, Zhang L, Zhang QF, Popov VL, Li C, Qu J, Li Q, Zhang YP, Hai R, Wu W, Wang Q, Zhan FX, Wang XJ, Kan B, Wang SW, Wan KL, Jing HQ, Lu JX, Yin WW, Zhou H, Guan XH, Liu JF, Bi ZQ, Liu GH, Ren J, Wang H, Zhao Z, Song JD, He JR, Wan T, Zhang JS, Fu XP, Sun LN, Dong XP, Feng ZJ, Yang WZ, Hong T, Zhang Y, Walker DH, Wang Y, Li DX 2011 Fever with thrombocytopenia associated with a novel bunyavirus in China. N Engl J Med 364:1523–1532. doi:10.1056/NEJMoa1010095. 21410387 PMC3113718

[6] Zhang YZ, He YW, Dai YA, Xiong Y, Zheng H, Zhou DJ, Li J, Sun Q, Luo XL, Cheng YL, Qin XC, Tian JH, Chen XP, Yu B, Jin D, Guo WP, Li W, Wang W, Peng JS, Zhang GB, Zhang S, Chen XM, Wang Y, Li MH, Li Z, Lu S, Ye C, de Jong MD, Xu J 2012 Hemorrhagic fever caused by a novel bunyavirus in China: pathogenesis and correlates of fatal outcome. Clin Infect Dis 54:527–533. doi:10.1093/cid/cir804. 22144540

[7] Kim KH, Yi J, Kim G, Choi SJ, Jun KI, Kim NH, Choe PG, Kim NJ, Lee JK, Oh MD 2013 Severe fever with thrombocytopenia syndrome, South Korea, 2012. Emerg Infect Dis 19:1892–1894. doi:10.3201/eid1911.130792. 24206586 PMC3837670

[8] Takahashi T, Maeda K, Suzuki T, Ishido A, Shigeoka T, Tominaga T, Kamei T, Honda M, Ninomiya D, Sakai T, Senba T, Kaneyuki S, Sakaguchi S, Satoh A, Hosokawa T, Kawabe Y, Kurihara S, Izumikawa K, Kohno S, Azuma T, Suemori K, Yasukawa M, Mizutani T, Omatsu T, Katayama Y, Miyahara M, Ijuin M, Doi K, Okuda M, Umeki K, Saito T, Fukushima K, Nakajima K, Yoshikawa T, Tani H, Fukushi S, Fukuma A, Ogata M, Shimojima M, Nakajima N, Nagata N, Katano H, Fukumoto H, Sato Y, Hasegawa H, Yamagishi T, Oishi K, Kurane I, Morikawa S, Saijo M 2014 The first identification and retrospective study of Severe Fever with Thrombocytopenia Syndrome in Japan. J Infect Dis 209:816–827. doi:10.1093/infdis/jit603. 24231186 PMC7107388

[9] McMullan LK, Folk SM, Kelly AJ, MacNeil A, Goldsmith CS, Metcalfe MG, Batten BC, Albariño CG, Zaki SR, Rollin PE, Nicholson WL, Nichol ST 2012 A new Phlebovirus associated with severe febrile illness in Missouri. N Engl J Med 367:834–841. doi:10.1056/NEJMoa1203378. 22931317

[10] Muehlenbachs A, Fata CR, Lambert AJ, Paddock CD, Velez JO, Blau DM, Staples JE, Karlekar MB, Bhatnagar J, Nasci RS, Zaki SR 2014 Heartland virus-associated death in Tennessee. Clin Infect Dis 59:845–850. doi:10.1093/cid/ciu434. 24917656 PMC4608028

[11] Xing Z, Schefers J, Schwabenlander M, Jiao Y, Liang M, Qi X, Li C, Goyal S, Cardona CJ, Wu X, Zhang Z, Li D, Collins J, Murtaugh MP 2013 Novel bunyavirus in domestic and captive farmed animals, Minnesota, USA. Emerg Infect Dis 19:1487–1489. doi:10.3201/eid1909.130165. 23966016 PMC5485073

[12] Kozuch O, Rajcáni J, Sekeyová M, Nosek J 1970 Uukuniemi virus in small rodents. Acta Virol 14:163–166. 4392850

[13] Saikku P 1973 Arboviruses in Finland. 3. Uukuniemi virus antibodies in human, cattle, and reindeer sera. Am J Trop Med Hyg 22:400–403. doi:10.4269/ajtmh.1973.22.400. 4735908

[14] Hubálek Z, Rudolf I 2012 Tick-borne viruses in Europe. Parasitol Res 111:9–36. doi:10.1007/s00436-012-2910-1. 22526290

[15] Swei A, Russell BJ, Naccache SN, Kabre B, Veeraraghavan N, Pilgard MA, Johnson BJ, Chiu CY 2013 The genome sequence of Lone Star virus, a highly divergent bunyavirus found in the Amblyomma americanum tick. PLoS One 8:e62083. doi:10.1371/journal.pone.0062083. 23637969 PMC3639253

[16] Gauci PJ, McAllister J, Mitchell IR, St George TD, Cybinski DH, Davis SS, Gubala AJ 2015 Hunter Island group phlebovirus in ticks, Australia. Emerg Infect Dis 21:2246–2248. doi:10.3201/eid2112.141303. 26583599 PMC4672409

[17] Mourya DT, Yadav PD, Basu A, Shete A, Patil DY, Zawar D, Majumdar TD, Kokate P, Sarkale P, Raut CG, Jadhav SM 2014 Malsoor virus, a novel bat phlebovirus, is closely related to severe fever with thrombocytopenia syndrome virus and Heartland virus. J Virol 88:3605–3609. doi:10.1128/JVI.02617-13. 24390329 PMC3957954

[18] Papa A, Kontana A, Tsioka K, Chaligiannis I, Sotiraki S 2016 Novel phleboviruses detected in ticks, Greece. Ticks Tick Borne Dis 7:690–693. doi:10.1016/j.ttbdis.2016.02.017. 26935112

[19] Tokarz R, Williams SH, Sameroff S, Sanchez Leon M, Jain K, Lipkin WI 2014 Virome analysis of Amblyomma americanum, Dermacentor variabilis, and Ixodes scapularis ticks reveals novel highly divergent vertebrate and invertebrate viruses. J Virol 88:11480–11492. doi:10.1128/JVI.01858-14. 25056893 PMC4178814

[20] Saito T, Gale M Jr 2008 Differential recognition of double-stranded RNA by RIG-I-like receptors in antiviral immunity. J Exp Med 205:1523–1527. doi:10.1084/jem.20081210. 18591413 PMC2442628

[21] Weber F, Wagner V, Rasmussen SB, Hartmann R, Paludan SR 2006 Double-stranded RNA is produced by positive-strand RNA viruses and DNA viruses but not in detectable amounts by negative-strand RNA viruses. J Virol 80:5059–5064. doi:10.1128/JVI.80.10.5059-5064.2006. 16641297 PMC1472073

[22] Son KN, Liang Z, Lipton HL 2015 Double-stranded RNA is detected by immunofluorescence analysis in RNA and DNA virus infections, including those by negative-stranded RNA viruses. J Virol 89:9383–9392. doi:10.1128/JVI.01299-15. 26136565 PMC4542381

[23] Hornung V, Ellegast J, Kim S, Brzózka K, Jung A, Kato H, Poeck H, Akira S, Conzelmann KK, Schlee M, Endres S, Hartmann G 2006 5′-Triphosphate RNA is the ligand for RIG-I. Science 314:994–997. doi:10.1126/science.1132505. 17038590

[24] Pichlmair A, Schulz O, Tan CP, Näslund TI, Liljeström P, Weber F, Reis e Sousa C 2006 RIG-I-mediated antiviral responses to single-stranded RNA bearing 5′-phosphates. Science 314:997–1001. doi:10.1126/science.1132998. 17038589

[25] Kawai T, Akira S 2007 Antiviral signaling through pattern recognition receptors. J Biochem 141:137–145. doi:10.1093/jb/mvm032. 17190786

[26] Randall RE, Goodbourn S 2008 Interferons and viruses: an interplay between induction, signalling, antiviral responses and virus countermeasures. J Gen Virol 89:1–47. doi:10.1099/vir.0.83391-0. 18089727

[27] Platanias LC 2005 Mechanisms of type-I- and type-II-interferon-mediated signalling. Nat Rev Immunol 5:375–386. doi:10.1038/nri1604. 15864272

[28] Bridgen A, Weber F, Fazakerley JK, Elliott RM 2001 Bunyamwera bunyavirus nonstructural protein NSs is a nonessential gene product that contributes to viral pathogenesis. Proc Natl Acad Sci U S A 98:664–669. doi:10.1073/pnas.98.2.664. 11209062 PMC14645

[29] Weber F, Bridgen A, Fazakerley JK, Streitenfeld H, Kessler N, Randall RE, Elliott RM 2002 Bunyamwera bunyavirus nonstructural protein NSs counteracts the induction of alpha/beta interferon. J Virol 76:7949–7955. doi:10.1128/JVI.76.16.7949-7955.2002. 12133999 PMC155133

[30] Santiago FW, Covaleda LM, Sanchez-Aparicio MT, Silvas JA, Diaz-Vizarreta AC, Patel JR, Popov V, Yu XJ, García-Sastre A, Aguilar PV 2014 Hijacking of RIG-I signaling proteins into virus-induced cytoplasmic structures correlates with the inhibition of type I interferon responses. J Virol 88:4572–4585. doi:10.1128/JVI.03021-13. 24478431 PMC3993744

[31] Ning YJ, Wang M, Deng M, Shen S, Liu W, Cao WC, Deng F, Wang YY, Hu Z, Wang H 2014 Viral suppression of innate immunity via spatial isolation of TBK1/IKKepsilon from mitochondrial antiviral platform. J Mol Cell Biol 6:324–337. doi:10.1093/jmcb/mju015. 24706939 PMC7107466

[32] Wu X, Qi X, Qu B, Zhang Z, Liang M, Li C, Cardona CJ, Li D, Xing Z 2014 Evasion of antiviral immunity through sequestering of TBK1/IKKepsilon/IRF3 into viral inclusion bodies. J Virol 88:3067–3076. doi:10.1128/JVI.03510-13. 24335286 PMC3957960

[33] Chaudhary V, Zhang S, Yuen KS, Li C, Lui PY, Fung SY, Wang PH, Chan CP, Li D, Kok KH, Liang M, Jin DY 2015 Suppression of type I and type III IFN signalling by NSs protein of severe fever with thrombocytopenia syndrome virus through inhibition of STAT1 phosphorylation and activation. J Gen Virol 96:3204–3211. doi:10.1099/jgv.0.000280. 26353965

[34] Ning YJ, Feng K, Min YQ, Cao WC, Wang M, Deng F, Hu Z, Wang H 2015 Disruption of type I interferon signaling by the nonstructural protein of severe fever with thrombocytopenia syndrome virus via the hijacking of STAT2 and STAT1 into inclusion bodies. J Virol 89:4227–4236. doi:10.1128/JVI.00154-15. 25631085 PMC4442386

[35] Rezelj VV, Överby AK, Elliott RM 2015 Generation of mutant Uukuniemi viruses lacking the nonstructural protein NSs by reverse genetics indicates that NSs is a weak interferon antagonist. J Virol 89:4849–4856. doi:10.1128/JVI.03511-14. 25673721 PMC4403475

[36] Elliott RM 2014 Orthobunyaviruses: recent genetic and structural insights. Nat Rev Microbiol 12:673–685. doi:10.1038/nrmicro3332. 25198140

[37] Sievers F, Wilm A, Dineen D, Gibson TJ, Karplus K, Li W, Lopez R, McWilliam H, Remmert M, Söding J, Thompson JD, Higgins DG 2011 Fast, scalable generation of high-quality protein multiple sequence alignments using Clustal Omega. Mol Syst Biol 7:539. doi:10.1038/msb.2011.75. 21988835 PMC3261699

[38] McWilliam H, Li W, Uludag M, Squizzato S, Park YM, Buso N, Cowley AP, Lopez R 2013 Analysis Tool Web services from the EMBL-EBI. Nucleic Acids Res 41:W597–W600. doi:10.1093/nar/gkt376. 23671338 PMC3692137

[39] Simons JF, Persson R, Pettersson RF 1992 Association of the nonstructural protein NSs of Uukuniemi virus with the 40S ribosomal subunit. J Virol 66:4233–4241. 1534850 10.1128/jvi.66.7.4233-4241.1992 PMC241227

[40] Yoneyama M, Suhara W, Fukuhara Y, Fukuda M, Nishida E, Fujita T 1998 Direct triggering of the type I interferon system by virus infection: activation of a transcription factor complex containing IRF-3 and CBP/p300. EMBO J 17:1087–1095. doi:10.1093/emboj/17.4.1087. 9463386 PMC1170457

[41] Panne D, McWhirter SM, Maniatis T, Harrison SC 2007 Interferon regulatory factor 3 is regulated by a dual phosphorylation-dependent switch. J Biol Chem 282:22816–22822. doi:10.1074/jbc.M703019200. 17526488

[42] Lin R, Heylbroeck C, Pitha PM, Hiscott J 1998 Virus-dependent phosphorylation of the IRF-3 transcription factor regulates nuclear translocation, transactivation potential, and proteasome-mediated degradation. Mol Cell Biol 18:2986–2996. doi:10.1128/MCB.18.5.2986. 9566918 PMC110678

[43] Fitzgerald KA, McWhirter SM, Faia KL, Rowe DC, Latz E, Golenbock DT, Coyle AJ, Liao SM, Maniatis T 2003 IKKepsilon and TBK1 are essential components of the IRF3 signaling pathway. Nat Immunol 4:491–496. doi:10.1038/ni921. 12692549

[44] Kawai T, Takahashi K, Sato S, Coban C, Kumar H, Kato H, Ishii KJ, Takeuchi O, Akira S 2005 IPS-1, an adaptor triggering RIG-I- and Mda5-mediated type I interferon induction. Nat Immunol 6:981–988. doi:10.1038/ni1243. 16127453

[45] Kowalinski E, Lunardi T, McCarthy AA, Louber J, Brunel J, Grigorov B, Gerlier D, Cusack S 2011 Structural basis for the activation of innate immune pattern-recognition receptor RIG-I by viral RNA. Cell 147:423–435. doi:10.1016/j.cell.2011.09.039. 22000019

[46] Onoguchi K, Onomoto K, Takamatsu S, Jogi M, Takemura A, Morimoto S, Julkunen I, Namiki H, Yoneyama M, Fujita T 2010 Virus-infection or 5′PPP-RNA activates antiviral signal through redistribution of IPS-1 mediated by MFN1. PLoS Pathog 6:e1001012. doi:10.1371/journal.ppat.1001012. 20661427 PMC2908619

[47] Clark K, Plater L, Peggie M, Cohen P 2009 Use of the pharmacological inhibitor BX795 to study the regulation and physiological roles of TBK1 and IkappaB kinase epsilon: a distinct upstream kinase mediates Ser-172 phosphorylation and activation. J Biol Chem 284:14136–14146. doi:10.1074/jbc.M109.000414. 19307177 PMC2682862

[48] Clark K, Peggie M, Plater L, Sorcek RJ, Young ER, Madwed JB, Hough J, McIver EG, Cohen P 2011 Novel cross-talk within the IKK family controls innate immunity. Biochem J 434:93–104. doi:10.1042/BJ20101701. 21138416

[49] Ma X, Helgason E, Phung QT, Quan CL, Iyer RS, Lee MW, Bowman KK, Starovasnik MA, Dueber EC 2012 Molecular basis of TANK-binding kinase 1 activation by transautophosphorylation. Proc Natl Acad Sci U S A 109:9378–9383. doi:10.1073/pnas.1121552109. 22619329 PMC3386122

[50] Kishore N, Huynh QK, Mathialagan S, Hall T, Rouw S, Creely D, Lange G, Caroll J, Reitz B, Donnelly A, Boddupalli H, Combs RG, Kretzmer K, Tripp CS 2002 IKK-I and TBK-1 are enzymatically distinct from the homologous enzyme IKK-2: comparative analysis of recombinant human IKK-i, TBK-1, and IKK-2. J Biol Chem 277:13840–13847. doi:10.1074/jbc.M110474200. 11839743

[51] Ho HH, Ivashkiv LB 2006 Role of STAT3 in type I interferon responses. Negative regulation of STAT1-dependent inflammatory gene activation. J Biol Chem 281:14111–14118. doi:10.1074/jbc.M511797200. 16571725

[52] Takeda K, Kaisho T, Yoshida N, Takeda J, Kishimoto T, Akira S 1998 Stat3 activation is responsible for IL-6-dependent T cell proliferation through preventing apoptosis: generation and characterization of T cell-specific Stat3-deficient mice. J Immunol 161:4652–4660. 9794394

[53] Oh HM, Yu CR, Lee Y, Chan CC, Maminishkis A, Egwuagu CE 2011 Autoreactive memory CD4^+^ T lymphocytes that mediate chronic uveitis reside in the bone marrow through STAT3-dependent mechanisms. J Immunol 187:3338–3346. doi:10.4049/jimmunol.1004019. 21832158 PMC3304102

[54] Yu CR, Dambuza IM, Lee YJ, Frank GM, Egwuagu CE 2013 STAT3 regulates proliferation and survival of CD8^+^ T cells: enhances effector responses to HSV-1 infection, and inhibits IL-10+ regulatory CD8^+^ T cells in autoimmune uveitis. Mediators Inflamm 2013:359674. doi:10.1155/2013/359674. 24204098 PMC3800609

[55] Banninger G, Reich NC 2004 STAT2 nuclear trafficking. J Biol Chem 279:39199–39206. doi:10.1074/jbc.M400815200. 15175343

[56] Fagerlund R, Mélen K, Kinnunen L, Julkunen I 2002 Arginine/lysine-rich nuclear localization signals mediate interactions between dimeric STATs and importin alpha 5. J Biol Chem 277:30072–30078. doi:10.1074/jbc.M202943200. 12048190

[57] Bouloy M, Janzen C, Vialat P, Khun H, Pavlovic J, Huerre M, Haller O 2001 Genetic evidence for an interferon-antagonistic function of Rift Valley fever virus nonstructural protein NSs. J Virol 75:1371–1377. doi:10.1128/JVI.75.3.1371-1377.2001. 11152510 PMC114043

[58] Stewart CE, Randall RE, Adamson CS 2014 Inhibitors of the interferon response enhance virus replication in vitro. PLoS One 9:e112014. doi:10.1371/journal.pone.0112014. 25390891 PMC4229124

[59] Liu MM, Lei XY, Yu H, Zhang JZ, Yu XJ 2017 Correlation of cytokine level with the severity of severe fever with thrombocytopenia syndrome. Virol J 14:6. doi:10.1186/s12985-016-0677-1. 28086978 PMC5237221

[60] Ryman KD, Klimstra WB, Nguyen KB, Biron CA, Johnston RE 2000 Alpha/beta interferon protects adult mice from fatal Sindbis virus infection and is an important determinant of cell and tissue tropism. J Virol 74:3366–3378. doi:10.1128/JVI.74.7.3366-3378.2000. 10708454 PMC111838

[61] Arimori Y, Nakamura R, Yamada H, Shibata K, Maeda N, Kase T, Yoshikai Y 2013 Type I interferon limits influenza virus-induced acute lung injury by regulation of excessive inflammation in mice. Antiviral Res 99:230–237. doi:10.1016/j.antiviral.2013.05.007. 23721943

[62] Ly HJ, Ikegami T 2016 Rift Valley fever virus NSs protein functions and the similarity to other bunyavirus NSs proteins. Virol J 13:118. doi:10.1186/s12985-016-0573-8. 27368371 PMC4930582

[63] Wuerth JD, Weber F 2016 Phleboviruses and the type I interferon response. Viruses 8:E174. doi:10.3390/v8060174. 27338447 PMC4926194

[64] Brennan B, Welch SR, Elliott RM 2014 The consequences of reconfiguring the ambisense S genome segment of Rift Valley fever virus on viral replication in mammalian and mosquito cells and for genome packaging. PLoS Pathog 10:e1003922. doi:10.1371/journal.ppat.1003922. 24550727 PMC3923772

[65] Cyr N, de la Fuente C, Lecoq L, Guendel I, Chabot PR, Kehn-Hall K, Omichinski JG 2015 A OmegaXaV motif in the Rift Valley fever virus NSs protein is essential for degrading p62, forming nuclear filaments and virulence. Proc Natl Acad Sci U S A 112:6021–6026. doi:10.1073/pnas.1503688112. 25918396 PMC4434773

[66] Yadani FZ, Kohl A, Préhaud C, Billecocq A, Bouloy M 1999 The carboxy-terminal acidic domain of Rift Valley Fever virus NSs protein is essential for the formation of filamentous structures but not for the nuclear localization of the protein. J Virol 73:5018–5025. 10233964 10.1128/jvi.73.6.5018-5025.1999 PMC112546

[67] Struthers JK, Swanepoel R 1982 Identification of a major non-structural protein in the nuclei of Rift Valley fever virus-infected cells. J Gen Virol 60:381–384. doi:10.1099/0022-1317-60-2-381. 7108491

[68] Léger P, Lara E, Jagla B, Sismeiro O, Mansuroglu Z, Coppée JY, Bonnefoy E, Bouloy M 2013 Dicer-2- and piwi-mediated RNA interference in Rift Valley fever virus-infected mosquito cells. J Virol 87:1631–1648. doi:10.1128/JVI.02795-12. 23175368 PMC3554164

[69] Le May N, Dubaele S, Proietti De Santis L, Billecocq A, Bouloy M, Egly JM 2004 TFIIH transcription factor, a target for the Rift Valley hemorrhagic fever virus. Cell 116:541–550. doi:10.1016/S0092-8674(04)00132-1. 14980221

[70] Kainulainen M, Habjan M, Hubel P, Busch L, Lau S, Colinge J, Superti-Furga G, Pichlmair A, Weber F 2014 Virulence factor NSs of Rift Valley fever virus recruits the F-box protein FBXO3 to degrade subunit p62 of general transcription factor TFIIH. J Virol 88:3464–3473. doi:10.1128/JVI.02914-13. 24403578 PMC3957945

[71] Le May N, Mansuroglu Z, Léger P, Josse T, Blot G, Billecocq A, Flick R, Jacob Y, Bonnefoy E, Bouloy M 2008 A SAP30 complex inhibits IFN-beta expression in Rift Valley fever virus infected cells. PLoS Pathog 4:e13. doi:10.1371/journal.ppat.0040013. 18225953 PMC2323286

[72] Kainulainen M, Lau S, Samuel CE, Hornung V, Weber F 2016 NSs virulence factor of Rift Valley fever virus engages the F-box proteins FBXW11 and beta-TRCP1 to degrade the antiviral protein kinase PKR. J Virol 90:6140–6147. doi:10.1128/JVI.00016-16. 27122577 PMC4907219

[73] Carlton-Smith C, Elliott RM 2012 Viperine, MTAP44, and protein kinase R contribute to the interferon-induced inhibition of Bunyamwera Orthobunyavirus replication. J Virol 86:11548–11557. doi:10.1128/JVI.01773-12. 22896602 PMC3486307

[74] Habjan M, Pichlmair A, Elliott RM, Overby AK, Glatter T, Gstaiger M, Superti-Furga G, Unger H, Weber F 2009 NSs protein of Rift Valley fever virus induces the specific degradation of the double-stranded RNA-dependent protein kinase. J Virol 83:4365–4375. doi:10.1128/JVI.02148-08. 19211744 PMC2668506

[75] Ikegami T, Narayanan K, Won S, Kamitani W, Peters CJ, Makino S 2009 Rift Valley fever virus NSs protein promotes post-transcriptional downregulation of protein kinase PKR and inhibits eIF2alpha phosphorylation. PLoS Pathog 5:e1000287. doi:10.1371/journal.ppat.1000287. 19197350 PMC2629125

[76] Kalveram B, Lihoradova O, Indran SV, Lokugamage N, Head JA, Ikegami T 2013 Rift Valley fever virus NSs inhibits host transcription independently of the degradation of dsRNA-dependent protein kinase PKR. Virology 435:415–424. doi:10.1016/j.virol.2012.09.031. 23063407 PMC3534933

[77] Kalveram B, Ikegami T 2013 Toscana virus NSs protein promotes degradation of double-stranded RNA-dependent protein kinase. J Virol 87:3710–3718. doi:10.1128/JVI.02506-12. 23325696 PMC3624217

[78] Gori Savellini G, Weber F, Terrosi C, Habjan M, Martorelli B, Cusi MG 2011 Toscana virus induces interferon although its NSs protein reveals antagonistic activity. J Gen Virol 92:71–79. doi:10.1099/vir.0.025999-0. 20861320

[79] Gori-Savellini G, Valentini M, Cusi MG 2013 Toscana virus NSs protein inhibits the induction of type I interferon by interacting with RIG-I. J Virol 87:6660–6667. doi:10.1128/JVI.03129-12. 23552410 PMC3676095

[80] Lihoradova OA, Indran SV, Kalveram B, Lokugamage N, Head JA, Gong B, Tigabu B, Juelich TL, Freiberg AN, Ikegami T 2013 Characterization of Rift Valley fever virus MP-12 strain encoding NSs of Punta Toro virus or sandfly fever Sicilian virus. PLoS Negl Trop Dis 7:e2181. doi:10.1371/journal.pntd.0002181. 23638202 PMC3630143

[81] Wu X, Qi X, Liang M, Li C, Cardona CJ, Li D, Xing Z 2014 Roles of viroplasm-like structures formed by nonstructural protein NSs in infection with severe fever with thrombocytopenia syndrome virus. FASEB J 28:2504–2516. doi:10.1096/fj.13-243857. 24599967

[82] Thomas NJ, Hunter DB, Atkinson CT 2007 Infectious diseases of wild birds, p 37 *In* Thomas NJ, Hunter DB, Atkinson CT (ed), Infectious diseases of wild birds. Wiley-Blackwell, Hoboken, NJ.

[83] Saikku P 1974 Passerine birds in the ecology of Uukuniemi virus. Med Biol 52:98–103. 4837432

[84] Varga ZT, Grant A, Manicassamy B, Palese P 2012 Influenza virus protein PB1-F2 inhibits the induction of type I interferon by binding to MAVS and decreasing mitochondrial membrane potential. J Virol 86:8359–8366. doi:10.1128/JVI.01122-12. 22674996 PMC3421771

[85] Varga ZT, Ramos I, Hai R, Schmolke M, García-Sastre A, Fernandez-Sesma A, Palese P 2011 The influenza virus protein PB1-F2 inhibits the induction of type I interferon at the level of the MAVS adaptor protein. PLoS Pathog 7:e1002067. doi:10.1371/journal.ppat.1002067. 21695240 PMC3111539

[86] Koshiba T, Yasukawa K, Yanagi Y, Kawabata S 2011 Mitochondrial membrane potential is required for MAVS-mediated antiviral signaling. Sci Signal 4:ra7. doi:10.1126/scisignal.2001147. 21285412

[87] Hu X, Ivashkiv LB 2009 Cross-regulation of signaling pathways by interferon-gamma: implications for immune responses and autoimmune diseases. Immunity 31:539–550. doi:10.1016/j.immuni.2009.09.002. 19833085 PMC2774226

[88] Takeda K, Kaisho T, Yoshida N, Takeda J, Kishimoto T, Akira S 2015 Correction: Stat3 activation is responsible for IL-6-dependent T cell proliferation through preventing apoptosis: generation and characterization of T cell-specific Stat3-deficient mice. J Immunol 194:3526. doi:10.4049/jimmunol.1500168. 25795790

[89] Deng B, Zhang S, Geng Y, Zhang Y, Wang Y, Yao W, Wen Y, Cui W, Zhou Y, Gu Q, Wang W, Wang Y, Shao Z, Wang Y, Li C, Wang D, Zhao Y, Liu P 2012 Cytokine and chemokine levels in patients with severe fever with thrombocytopenia syndrome virus. PLoS One 7:e41365. doi:10.1371/journal.pone.0041365. 22911786 PMC3404083

[90] Jin C, Liang M, Ning J, Gu W, Jiang H, Wu W, Zhang F, Li C, Zhang Q, Zhu H, Chen T, Han Y, Zhang W, Zhang S, Wang Q, Sun L, Liu Q, Li J, Wang T, Wei Q, Wang S, Deng Y, Qin C, Li D 2012 Pathogenesis of emerging severe fever with thrombocytopenia syndrome virus in C57/BL6 mouse model. Proc Natl Acad Sci U S A 109:10053–10058. doi:10.1073/pnas.1120246109. 22665769 PMC3382536

[91] Hilton L, Moganeradj K, Zhang G, Chen YH, Randall RE, McCauley JW, Goodbourn S 2006 The NPro product of bovine viral diarrhea virus inhibits DNA binding by interferon regulatory factor 3 and targets it for proteasomal degradation. J Virol 80:11723–11732. doi:10.1128/JVI.01145-06. 16971436 PMC1642611

[92] Oker-Blom N, Salminen A, Brummer-Korvenkontio M, Kaeaeriaeinen L, Weckstroem P 1964 Isolation of some viruses other than typical tick-borne encephalitis viruses from Ixodes ricinus ticks in Finland. Ann Med Exp Biol Fenn 42:109–112. 14229542

[93] Brennan B, Li P, Zhang S, Li A, Liang M, Li D, Elliott RM 2015 Reverse genetics system for severe fever with thrombocytopenia syndrome virus. J Virol 89:3026–3037. doi:10.1128/JVI.03432-14. 25552716 PMC4337530

[94] Katz A, Freiberg AN, Backström V, Schulz AR, Mateos A, Holm L, Pettersson RF, Vaheri A, Flick R, Plyusnin A 2010 Oligomerization of Uukuniemi virus nucleocapsid protein. Virol J 7:187. doi:10.1186/1743-422X-7-187. 20698970 PMC2925374

[95] King P, Goodbourn S 1998 STAT1 is inactivated by a caspase. J Biol Chem 273:8699–8704. doi:10.1074/jbc.273.15.8699. 9535846

[96] Yoneyama M, Suhara W, Fukuhara Y, Sato M, Ozato K, Fujita T 1996 Autocrine amplification of type I interferon gene expression mediated by interferon stimulated gene factor 3 (ISGF3). J Biochem 120:160–169. doi:10.1093/oxfordjournals.jbchem.a021379. 8864859

[97] Prins KC, Cárdenas WB, Basler CF 2009 Ebola virus protein VP35 impairs the function of interferon regulatory factor-activating kinases IKKepsilon and TBK-1. J Virol 83:3069–3077. doi:10.1128/JVI.01875-08. 19153231 PMC2655579

[98] Park MS, Shaw ML, Muñoz-Jordan J, Cros JF, Nakaya T, Bouvier N, Palese P, García-Sastre A, Basler CF 2003 Newcastle disease virus (NDV)-based assay demonstrates interferon-antagonist activity for the NDV V protein and the Nipah virus V, W, and C proteins. J Virol 77:1501–1511. doi:10.1128/JVI.77.2.1501-1511.2003. 12502864 PMC140815

[99] van Knippenberg I, Elliott RM 2015 Flexibility of bunyavirus genomes: creation of an Orthobunyavirus with an ambisense S segment. J Virol 89:5525–5535. doi:10.1128/JVI.03595-14. 25740985 PMC4442540

[100] Waterhouse AM, Procter JB, Martin DM, Clamp M, Barton GJ 2009 Jalview version 2—a multiple sequence alignment editor and analysis workbench. Bioinformatics 25:1189–1191. 19151095 10.1093/bioinformatics/btp033 PMC2672624

