# Supplementary figures and images for: Differential Antagonism of Human Innate Immune Responses by Tick-Borne Phlebovirus Nonstructural Proteins
Source: mSphere. 2017 Jun 28;2(3):e00234-17. doi: 10.1128/mSphere.00234-17 (PMC5489658; doi:10.1128/mSphere.00234-17)

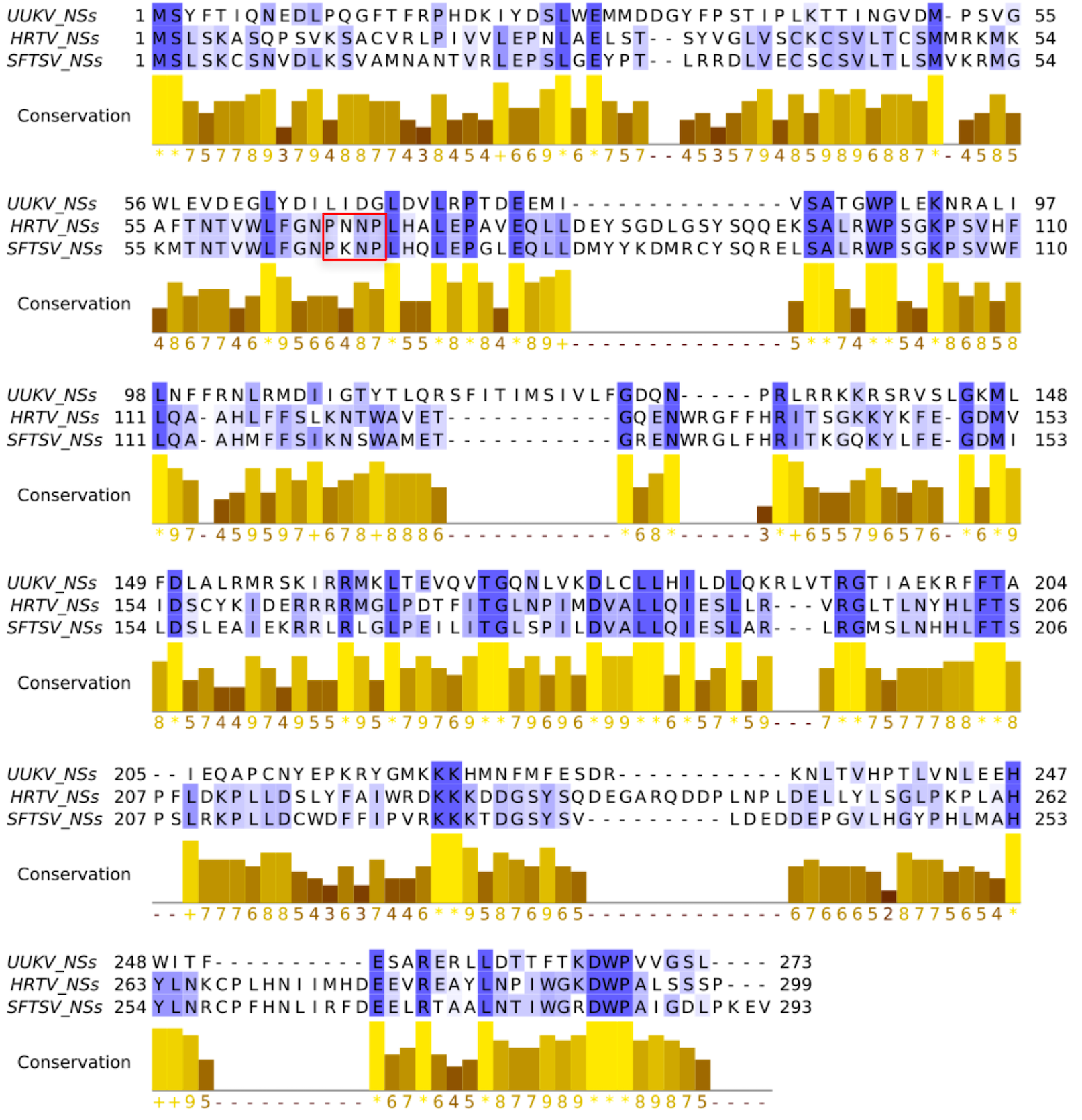

Supplement: FIG S1 [file sph003172310sf1.tif]

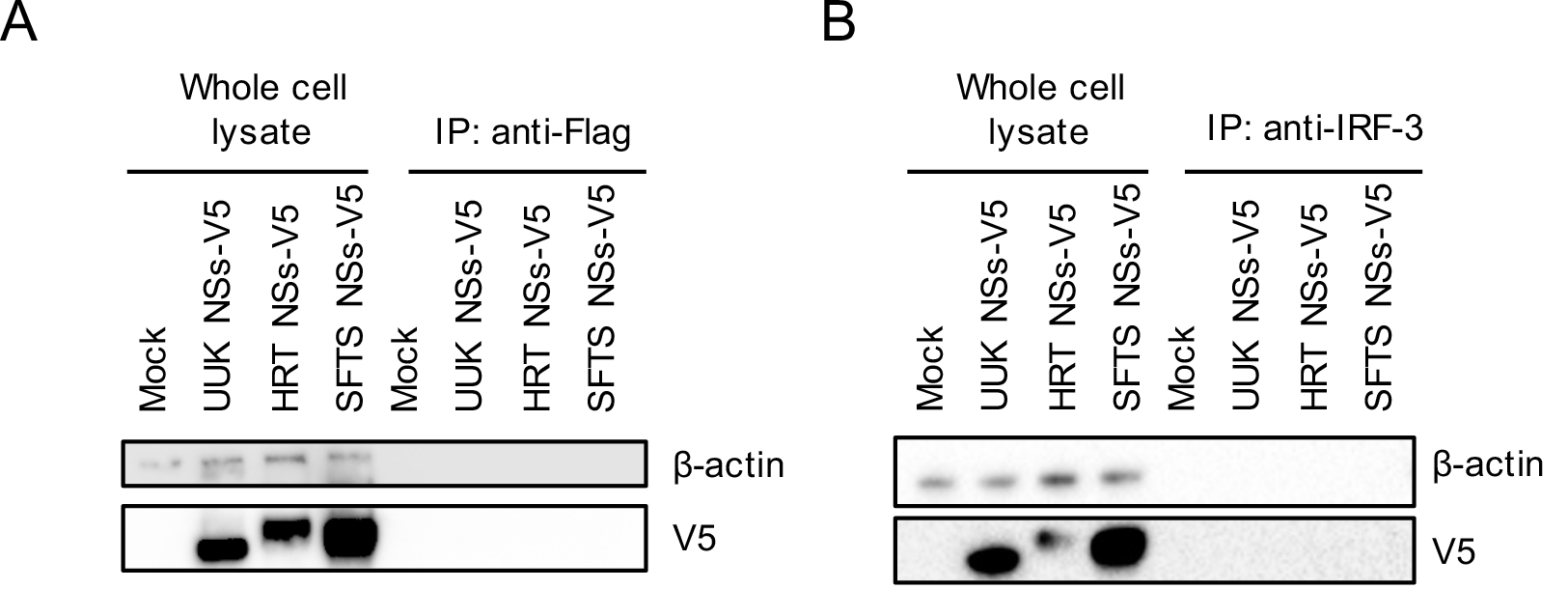

Supplement: FIG S2 [file sph003172310sf2.tif]

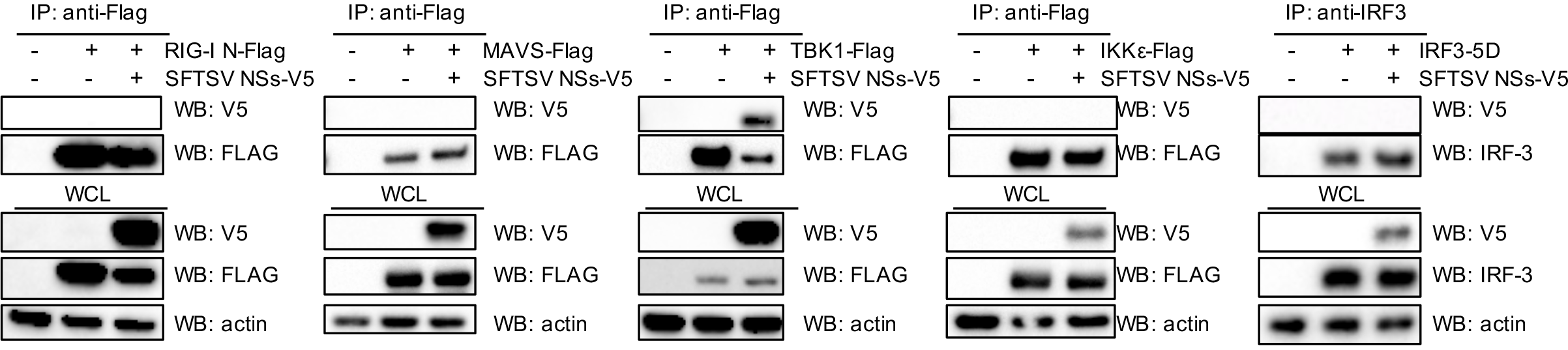

Supplement: FIG S3 [file sph003172310sf3.tif]

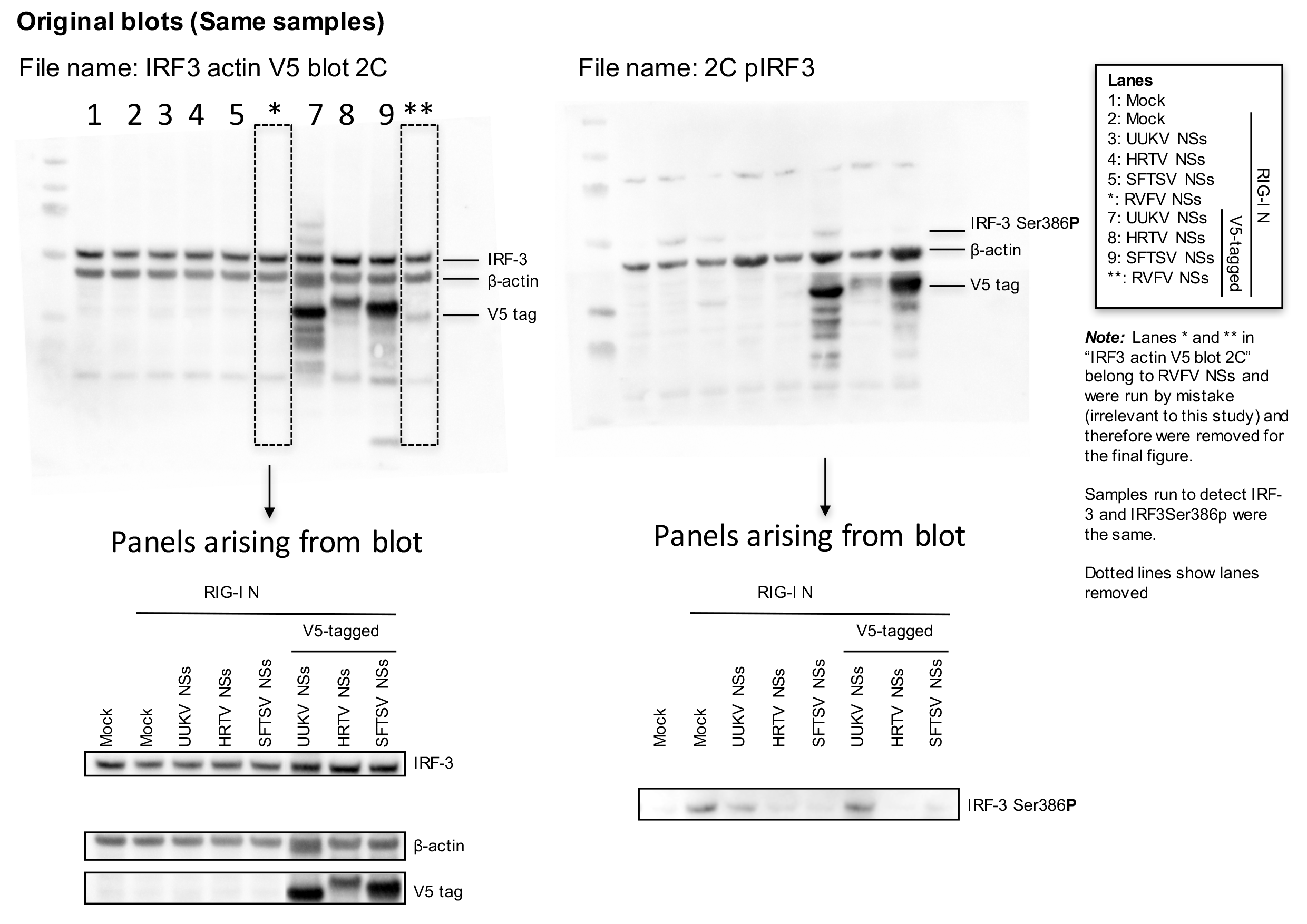

Supplement: FIG S4 [file sph003172310sf4.tif]
